# Plasticity of *Escherichia coli* cell wall metabolism promotes fitness and antibiotic resistance across environmental conditions

**DOI:** 10.7554/eLife.40754

**Published:** 2019-04-09

**Authors:** Elizabeth A Mueller, Alexander JF Egan, Eefjan Breukink, Waldemar Vollmer, Petra Anne Levin

**Affiliations:** 1 Department of BiologyWashington University in St. LouisSt. LouisUnited States; 2 Centre for Bacterial Cell Biology, Institute for Cell and Molecular BiosciencesNewcastle UniversityNewcastle upon TyneUnited Kingdom; 3 Membrane Biochemistry and Biophysics, Department of Chemistry, Faculty of ScienceUtrecht UniversityUtrechtNetherlands; Massachusetts Institute of TechnologyUnited States; National Institute of Child Health and Human DevelopmentUnited States

**Keywords:** redundancy, penicillin binding proteins, pH, Peptidoglycan, beta-lactam antibiotics, *E. coli*

## Abstract

Although the peptidoglycan cell wall is an essential structural and morphological feature of most bacterial cells, the extracytoplasmic enzymes involved in its synthesis are frequently dispensable under standard culture conditions. By modulating a single growth parameter—extracellular pH—we discovered a subset of these so-called ‘redundant’ enzymes in *Escherichia coli* are required for maximal fitness across pH environments. Among these pH specialists are the class A penicillin binding proteins PBP1a and PBP1b; defects in these enzymes attenuate growth in alkaline and acidic conditions, respectively. Genetic, biochemical, and cytological studies demonstrate that synthase activity is required for cell wall integrity across a wide pH range and influences pH-dependent changes in resistance to cell wall active antibiotics. Altogether, our findings reveal previously thought to be redundant enzymes are instead specialized for distinct environmental niches. This specialization may ensure robust growth and cell wall integrity in a wide range of conditions.

**Editorial note:** This article has been through an editorial process in which the authors decide how to respond to the issues raised during peer review. The Reviewing Editor's assessment is that all the issues have been addressed (see decision letter).

## Introduction

The growth and survival of single-celled organisms relies on their ability to adapt to rapidly changing environmental conditions. A commensal, pathogen, and environmental contaminant, *Escherichia coli* occupies and grows in diverse environmental niches, including the gastrointestinal tract, bladder, freshwater, and soil. In the laboratory, the bacterium’s flexibility in growth requirements is reflected in robust proliferation across a wide range of temperature, salt, osmotic, pH, oxygenation, and nutrient conditions (Ingraham and Marr, 1996).

The physiological adaptations that permit growth and survival across environmental conditions are not yet well understood, particularly for extracytoplasmic processes. Due to the discrepancy in permeability between the plasma and outer membrane (Rosenbusch, 1990), the periplasmic space of Gram-negative bacteria is sensitive to chemical and physical perturbations, including changes in salt, ionic strength, osmolality, and pH. Notably, upon mild environmental acidification, the periplasm assumes the pH of the extracellular media (Slonczewski et al., 1981; Wilks and Slonczewski, 2007). Although mechanisms that contribute to cytoplasmic pH homeostasis have been described in detail (Castanie-Cornet et al., 1999; Castanié-Cornet et al., 2010), comparatively little is known about the quality control mechanisms that preserve proper folding, stability, and activity of key proteins in the periplasm.

The peptidoglycan (PG) cell wall and its synthetic machinery are among the fundamental constituents of the periplasm that must be preserved across growth conditions. Essential for viability among most bacteria, PG is composed of glycan strands of repeating *N-*acetylglucosamine and *N-*acetylmuramic acid disaccharide units crosslinked at peptide stems (Vollmer et al., 2008). Beyond providing a force necessary to resist turgor pressure, the cell wall maintains cell shape, and components of the cell envelope serve as a major interface for cell-cell and cell-host interactions (Typas et al., 2012; McDonald et al., 2005). As an essential process, PG synthesis is also the principle target of several classes of antibacterial agents, including β-lactam (e.g. penicillin) and glycopeptide (e.g. vancomycin) antibiotics.

PG precursors are assembled in the cytosol and translocated across the inner membrane into the periplasm, where cell wall synthases construct the PG network through a series of glycosyltransferase (glycan polymerizing) and transpeptidase (peptide crosslinking) reactions. PG synthases include bifunctional class A penicillin binding proteins (PBPs), as well as monofunctional transpeptidases (class B PBPs) and monofunctional glycosyltransferases of the shape, elongation, division, and sporulation (SEDS) protein families (Sauvage et al., 2008; Meeske et al., 2016; Cho et al., 2016; Taguchi et al., 2019). LD-transpeptidases synthesize non-canonical LD-crosslinks between peptide stems. They are predominately active during PG remodeling during stationary phase growth in *E. coli* (Pisabarro et al., 1985; Magnet et al., 2007) and are required under severe envelope stress (Morè et al., 2019). In addition to synthases, a series of periplasmic cell wall hydrolases and autolysins—including DD-carboxypeptidases, DD and LD-endopeptidases, lytic transglycosylases, and amidases—are required to accommodate nascent strand insertion for expansion of the PG network, create substrate binding sites, and separate cells during the final stages of cytokinesis (Typas et al., 2012). These enzymes may also play a role activating synthases to ensure cell wall integrity (Lai et al., 2017).

The periplasmic steps of PG synthesis and remodeling exhibit high levels of enzymatic redundancy, the function of which remains unclear. While the cytoplasmic steps of PG precursor biogenesis in *E. coli* have nearly a 1:1 stochiometric ratio between reactions and enzymes (12 reactions: 14 enzymes), over 36 enzymes can carry out the nine reactions that take place in the periplasm (Pazos et al., 2017). Moreover, with the exception of the SEDS glycosyltransferase/bPBP pairs RodA/PBP2 and FtsW/PBP3 required for lateral expansion of the cell wall and cell division, respectively (Meeske et al., 2016; Cho et al., 2016), the remaining periplasmic cell wall enzymes appear to be nonessential. Inactivation of an individual enzyme—and in some cases, even multiple enzymes in the same class—often fails to confer discernable growth or morphological phenotypes under standard culture conditions (Singh et al., 2012; Nelson and Young, 2000; Heidrich et al., 2002; Suzuki et al., 1978; Heidrich et al., 2001). Although technological breakthroughs have aided in the identification of cell wall metabolic genes encoding proteins with similar functions (e.g. Peters et al., 2016a), elucidating the potential fitness benefit to redundancy has proven challenging.

One model to account for the apparent redundancy of periplasmic cell wall proteins is that enzymes within a given class may be specialists for distinct environmental niches, thereby allowing bacteria to cope with the diverse chemical and physical properties that might affect protein stability and function in this compartment (Pazos et al., 2017). In support of this hypothesis, several groups have identified cell wall enzymes that have increased activity in acidic media. Peters and colleagues demonstrated that *E. coli* carboxypeptidase PBP6b plays a key role in maintenance of cell morphology during growth at pH 5.0 (Peters et al., 2016b), while Castanheira *et al.* identified a PBP3 homolog in *Salmonella* Typhimurium that is preferentially involved in septation at low pH (Castanheira et al., 2017). Similarly, the lytic transglycosylase MltA exhibits maximal activity in acidic conditions in vitro (van Straaten et al., 2005), although whether this property is relevant in vivo remains unknown.

In light of these findings, we hypothesized that loss of an enzyme specialized for a particular environmental niche may produce a condition-specific growth defect through impaired cell wall integrity, allowing us to take a systems-level approach to identifying enzymes with differential roles in growth in vivo. In screening 32 mutants across six classes of nonessential periplasmic cell wall enzymes, we determined that a subset of these enzymes is differentially required for fitness across pH environments. We find that disruptions in the activity of cell wall synthases PBP1a and PBP1b conferred fitness defects in opposing pH ranges that can be attributed in part to pH-dependent differences in enzymatic activity. We further demonstrate that synthase specialization has consequences for intrinsic resistance to β-lactam antibiotics in nonstandard growth conditions.

## Results

### Identification of pH specialist cell wall synthases and hydrolases

To determine the contribution of individual cell wall enzymes to pH-dependent growth, we cultured strains harboring deletions in genes encoding each of three class A PBPs, six LD-transpeptidases, five carboxypeptidases, four amidases, nine lytic glycosyltransferases, and six endopeptidases to mid-exponential phase (OD_600_ ~0.2–0.6) in buffered LB media (pH 6.9) then sub-cultured them into fresh LB buffered to pH 4.8, 6.9, or 8.2 for growth rate analysis. These pH values were chosen as representative, physiologically relevant conditions *E. coli* cells encounter in the lower GI tract (pH 5–9) or urine (pH 4.5–8) (Watson et al., 1972; Henderson and Palmer, 1912). Preliminary hits were identified by a significant (>5%) decrease in early exponential phase (OD_600_0.005–0.1) mass doublings per hour (DPH) in at least one pH condition compared to the parental strain. Representative growth curves and fits are presented in Figure 1—figure supplement 1. Mutants exhibiting significant growth defects at one or more pH values were re-tested across an expanded set of pH conditions for validation; those that displayed consistent growth defects across a discrete range of pH values were classified as pH-sensitive mutants (Figure 1—figure supplement 2; Figure 2; Supplementary file 3).

**Figure 1.**
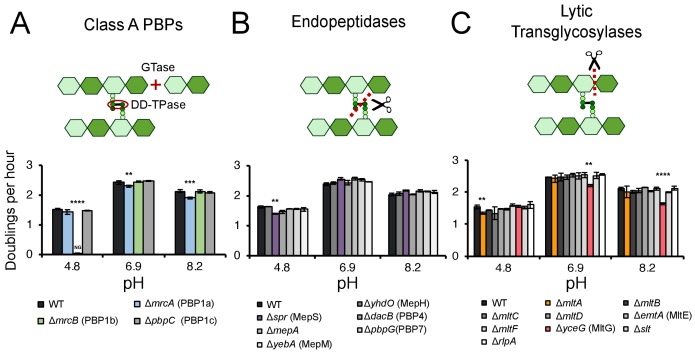
Identification of pH specialist cell wall enzymes. Mutants in genes encoding for non-essential class A PBPs (**A**), endopeptidases (**B**), and lytic transglycosylases (**C**) were screened for growth defects compared to the parental strain in LB media buffered to pH 4.8, 6.9, or 8.2. Bars depict mean mass doublings per hour ±SD of three independent biological replicates. NG denotes ‘no growth’ observed throughout the course of the experiment (20 hr). Cartoons depict enzymatic activity of the indicated enzyme class with GTase and DD-TPase referring to glycosyltransferase and DD-transpeptidase activity, respectively. Asterisks denote a significant >5% growth defect as determined by a one-way ANOVA corrected for multiple comparisons as follows: **, p<0.01; ***, p<0.001; ****p<0.0001. Mean ± SD values for mutants in this figure and all additional mutants tested can be viewed in Supplementary file 3. Representative growth curves, fits, and source data from the class A PBP mutants can be viewed in Figure 1—figure supplement 1 and Figure 1—source datas 1–3. 10.7554/eLife.40754.006Figure 1—source data 1.Representative source data for class A PBP mutants at pH 4.8.Supports Figure 1 and Figure 1—figure supplement 1. This data was used to generate growth curves, fits, and fit statistics in Figure 1—figure supplement 1. 10.7554/eLife.40754.007Figure 1—source data 2.Representative source data for class A PBP mutants at pH 6.9.Supports Figure 1 and Figure 1—figure supplement 1. This data was used to generate growth curves, fits, and fit statistics in Figure 1—figure supplement 1. 10.7554/eLife.40754.008Figure 1—source data 3.Representative source data for class A PBP mutants at pH 8.2.Supports Figure 1 and Figure 1—figure supplement 1. This data was used to generate growth curves, fits, and fit statistics in Figure 1—figure supplement 1.

**Figure 1—figure supplement 1.**
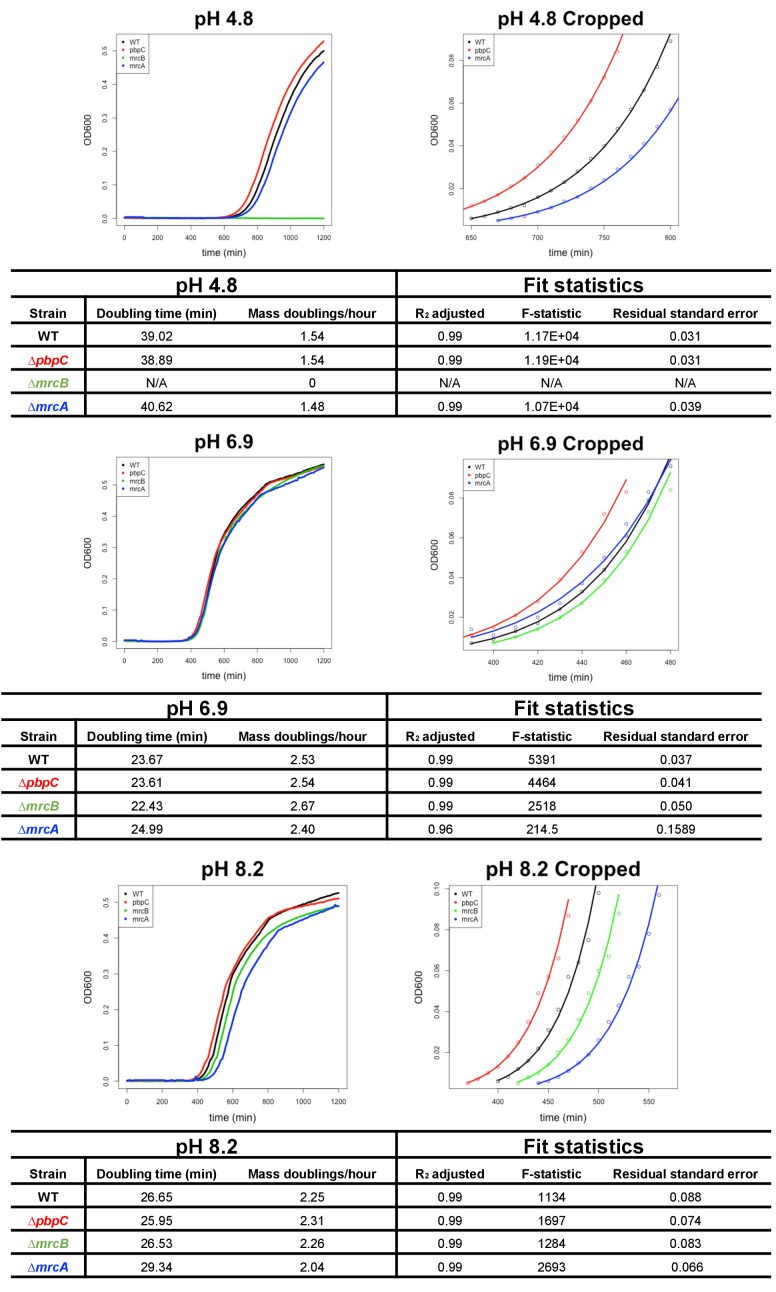
Growth rate determination pipeline. Growth rate (mass doublings per hour) for each mutant was assessed in buffered LB (various pH values) during growth in a 96-well microtiter plate at 37°C with aeration. Cells were inoculated into the plate at 1 × 10^3^ CFU/mL, and OD_600_ was measured every 10 min for 20 hr. From this data, growth curves were plotted and cropped to early exponential phase (OD_600_ values between 0.005–0.1). The resulting data used to determine DPH by least-squares fitting in R. Full and cropped growth curves, including best fit lines and fit statistics, of a single replicate for cells defective for each of the three class A PBPs and the parental strain at pH 4.8, 6.9, and 8.2 are shown here as representative examples. Fits with R^2 ^values < 0.95 were excluded from further analysis. Source data presented here can be found in Figure 1—source data 1–3, and a representative script is included in Supplementary file 7.

**Figure 1—figure supplement 2.**
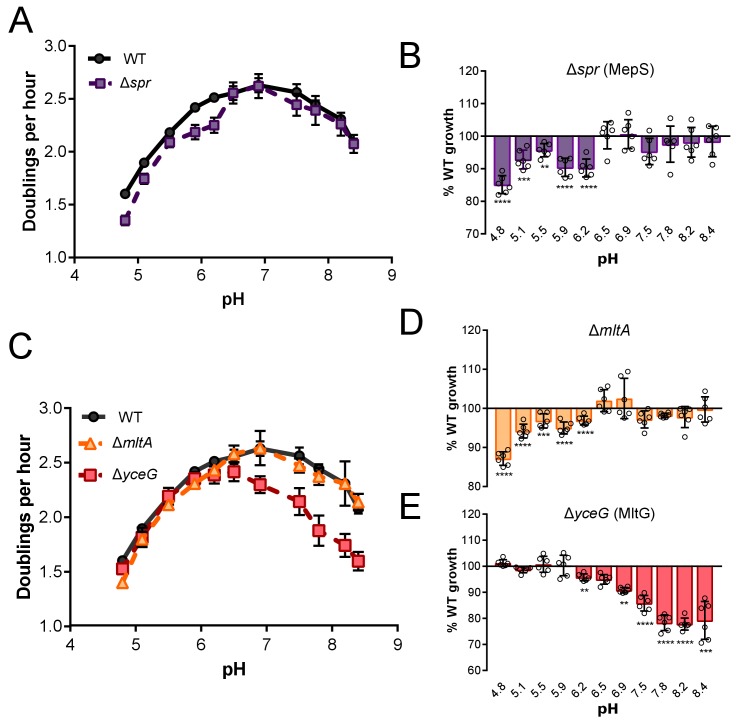
Growth rate determination of hits across an expanded set of pH conditions. Mean mass doublings per hour and transformed percent parental growth for endopeptidase mutant Δ*spr* (EAM1032; **A and B**) and lytic transglycosylase mutants Δ*mltA* and Δ*yceG* (EAM790 and EAM798; **C–E**). Significance was determined by an unpaired t-test corrected for multiple comparisons using the Holm-Sidak method. Error bars represent SD from six independent biological replicates. Asterisks denote significance as follows: **, p<0.01; ***, p<0.001; ****p<0.0001.

**Figure 1—figure supplement 3.**
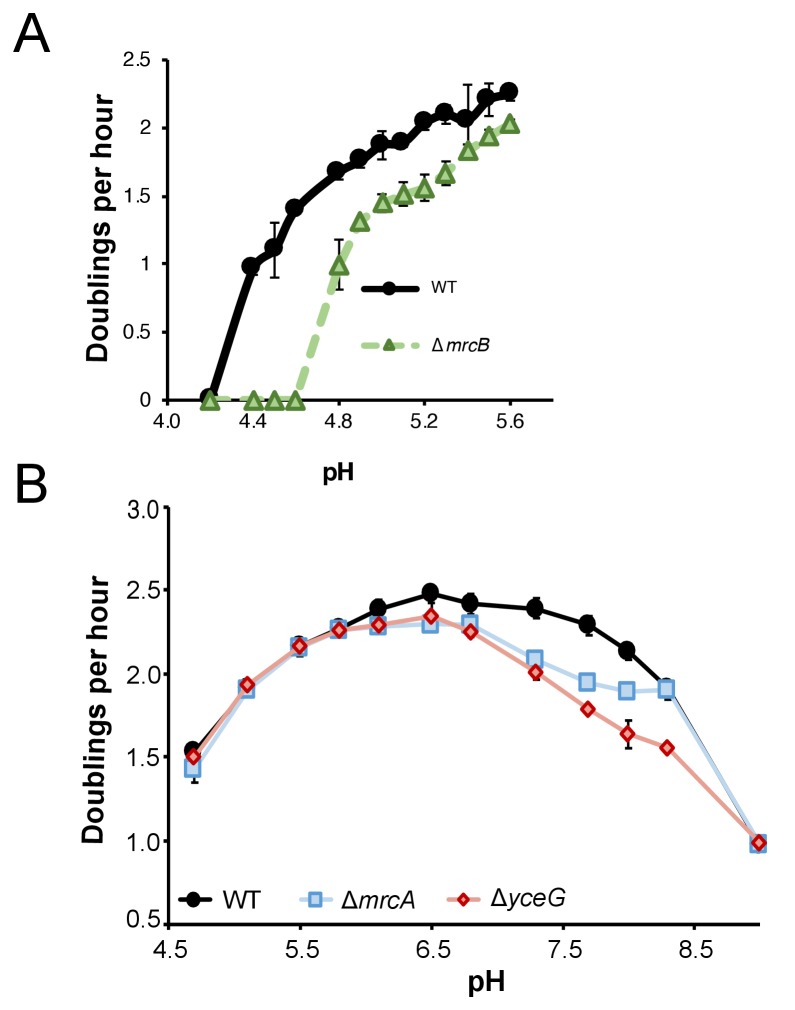
Growth of class A PBP mutants in extreme pH conditions. (**A**) Mean mass doublings per hour of ∆*mrcB* (PBP1b; EAM546) and the parental strain (MG1655) in LB media buffered from pH 4.2–5.8. Strains were pre-cultured in acidic media (LB +MMT, pH 5.5). (**B**) Mean mass doublings per hour of ∆*mrcA* (PBP1a; EAM543), ∆*yceG* (EAM798), and the parental strain (MG1655) from pH 4.8–9.0. Error bars represent SD from three biological replicates for both A and B.

**Figure 2.**
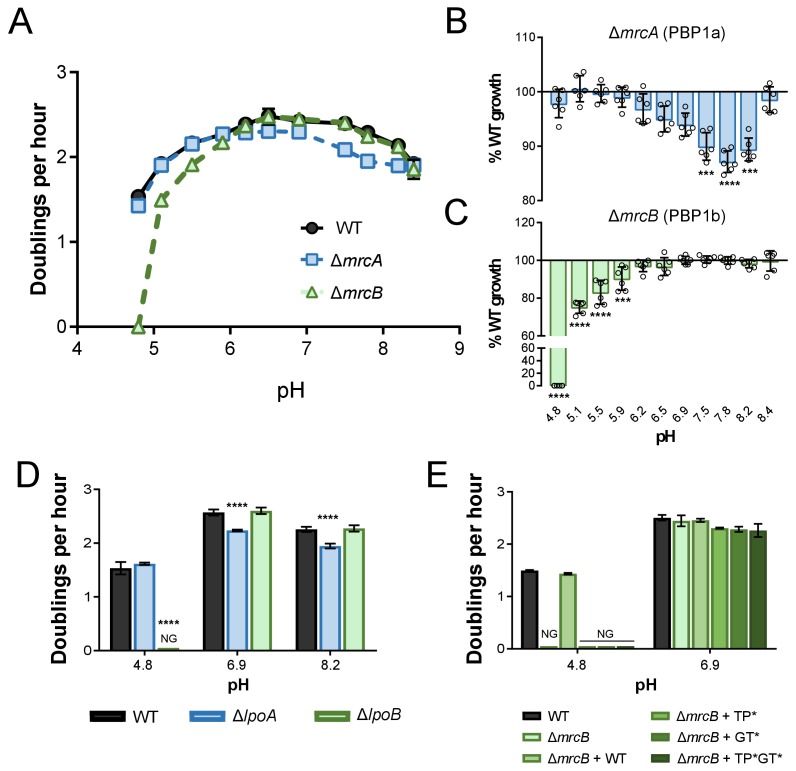
pH-dependent growth requires class A PBP activity. (**A–C**) Mean mass doublings per hour and transformed percent parental growth for ∆*mrcA* (PBP1a; EAM543) and ∆*mrcB* (PBP1b; EAM546) deletions compared to parental strain (MG1655) in LB media buffered from pH 4.8–8.4 Significance was determined by an unpaired t-test corrected for multiple comparisons using the Holm-Sidak method. Error bars represent SD from six independent biological replicates. (**D**) Growth rate analysis of cells defective for LpoA (EAM657) and LpoB (EAM659) cultured in buffered LB at pH 4.8, 6.9, or 8.0. Bars represent mean mass doublings per hour ± SD from three independent biological replicates. Asterisks denote significance as determined by a one-way ANOVA corrected for multiple comparisons as follows: ****, p<0.0001. Growth of these mutants across an expanded set of pH conditions can be viewed in Figure 2—figure supplement 2. (**D**) Complementation analysis of PBP1b variants synthesized from a plasmid (pUM1Bα, pUM1Bα*, pUM1BTG*α, or pUM1BTG*α*) and induced with 5 μM IPTG in the Δ*mrcB* (EAM696) background in buffered LB at pH 4.8 and 6.9. Bars represent mean mass doublings per hour ± SD from three independent biological replicates. NG denotes ‘no growth’ observed throughout the course of the experiment (20 hr).

**Figure 2—figure supplement 1.**
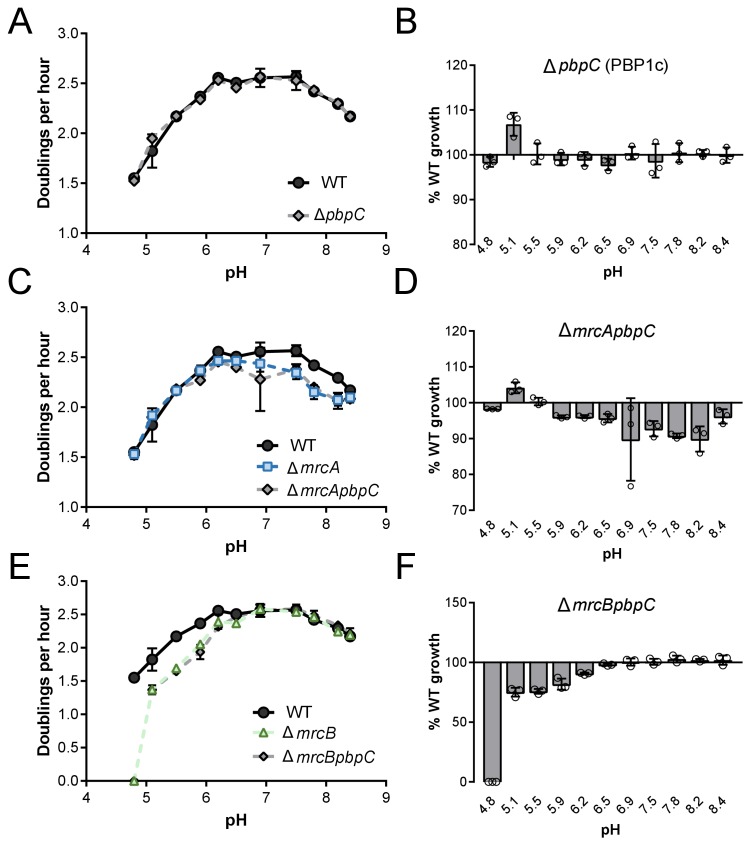
Growth rate analysis of strains defective for PBP1c. Mean mass doublings per hour and transformed percent parental growth for ∆*pbpC* (EAM694; **A, B**), ∆*mrcApbpC* (EAM1137; **C, D**), and ∆*mrcBpbpC* (EAM1139; **E, F**) compared their parental strains in LB media buffered from pH 4.8–8.4. Error bars indicate SD of three independent biological replicates.

**Figure 2—figure supplement 2.**
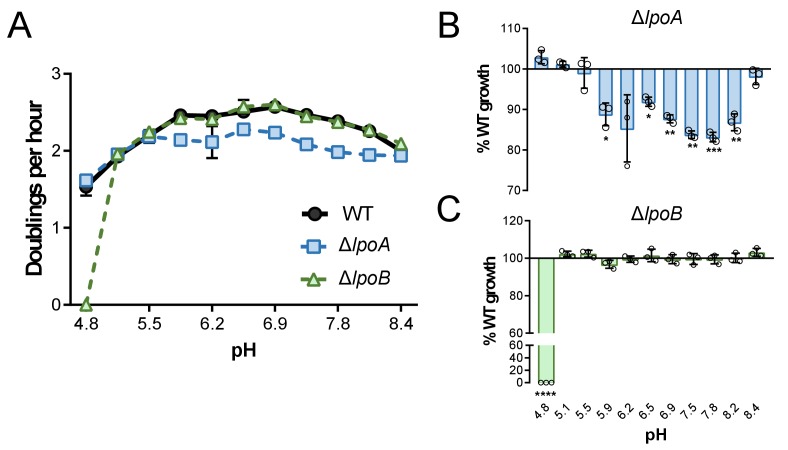
Growth rate determination of *lpo* mutants across an expanded set of pH conditions. Mean mass doublings per hour and transformed percent parental growth for ∆*lpoA* (EAM657) and ∆*lpoB* (EAM659) mutants compared to the parental strain (MG1655) from pH 4.8–8.4. Significance was determined by an unpaired t-test corrected for multiple comparisons using the Holm-Sidak method. Error bars represent SD from six independent biological replicates. Asterisks denote significance as follows: *, p<0.05; **, p<0.01; ***, p<0.001; ****, p<0.0001.

Collectively, five mutants met these stringent criteria and displayed significant pH-dependent reductions in DPH. We observed both acid-sensitive and alkaline-sensitive mutants across three enzymatic classes. Strikingly, loss of the bifunctional synthase PBP1b (*mrcB*) abolished growth at pH 4.8—over half a pH unit lower than the growth restrictive condition for the parental strain (Figure 1A; Figure 1—figure supplement 3A). Growth of the Δ*mrcB* mutant was also significantly attenuated (10–25% defect in DPH) at pH values between 5.1–5.9 but was indistinguishable from the parental strain in neutral and alkaline pH (Figure 2A,C). Pre-conditioning the mutant in acidic media (pH 5.5) did not abrogate the growth rate defect (Figure 1—figure supplement 3A), indicating that steady-state pH—rather than pH shock—underlies the defect in DPH. Mutants defective in production of lytic transglycosylase MltA and endopeptidase MepS (*spr*) also exhibited a specific, albeit less severe, defect in DPH in acidic media: their growth was attenuated by 5–15% compared to wild type cells at pH values at or below 6.2 (Figure 1B,C; Figure 1—figure supplement 2). Consistent with a role in acid tolerance, MltA was previously shown to have elevated enzymatic activity in acidic conditions in vitro (van Straaten et al., 2005), and cells defective for PBP1b or MepS production exhibit reduced colony growth on acidic agarose plates (Nichols et al., 2011).

We also identified two alkaline-sensitive mutants. Loss of the bifunctional synthase PBP1a (*mrcA*) and the lytic transglycosylase MltG (*yceG*) impaired, but did not abolish, growth specifically in neutral and alkaline media (Figure 1A,C; Figure 1—figure supplement 2; Figure 2A,C). Loss of MltG was associated with a greater magnitude and range of growth impairment (pH 6.2–8.4 compared to pH 6.5–8.2 for Δ*mrcA*). Both mutants’ growth was restored to wild-type levels in acidic media (pH <6.0) and at pH 9.0 (Figure 1—figure supplement 3B).

Individual deletions in the genes encoding for the six LD-transpeptidases, five carboxypeptidases, and four amidases failed to confer any pH-dependent defects in DPH at any pH tested, consistent with either a limited role of these enzymes in exponential phase growth (Pisabarro et al., 1985; Magnet et al., 2007) or additional layers of redundancy (Supplementary file 3).

### Class A PBP activity ensures fitness across a wide pH range

Given their opposing impact on DPH under acidic and alkaline conditions, we elected to focus further efforts on understanding the contribution of the bifunctional class A PBPs PBP1a and PBP1b to growth across a range of pH conditions. An accumulating body of evidence suggests the class A PBPs play overlapping, and potentially redundant, roles in PG synthesis during growth in standard culture conditions (i.e. nutrient rich, neutral pH growth medium aerated at 37°C) (Cho et al., 2016; Yousif et al., 1985). Indeed, *E. coli* requires at least one of these enzymes for viability during growth in standard culture conditions (Suzuki et al., 1978).

Based on their disparate pH-dependent growth defects, we hypothesized that PBP1a and PBP1b are specialized synthases whose activity is essential for maximal growth in distinct pH environments. Consistent with this model, cells defective in PBP1a (Δ*mrcA*) and PBP1b (Δ*mrcB*) displayed defects in DPH at discrete, non-overlapping pH ranges (Figure 2A–C). Loss of PBP1c (*pbpC*), a third class A PBP with an unclear role in cell wall metabolism (Schiffer and Höltje, 1999), did not result in a defect in DPH at any pH tested alone or combination with cells defective for PBP1a or PBP1b (Figure 2—figure supplement 1), indicating this enzyme does not play a role in pH-dependent growth under the conditions tested here.

We next sought to test whether PBP transpeptidase and/or glycosyltransferase activity were required for fitness across pH conditions, as opposed to an indirect, structural role for these enzymes in the formation of cell wall synthesis complexes (Müller et al., 2007; Bertsche et al., 2006). To test this, we took advantage of two sets of mutants: 1) deletions in *lpoA* and *lpoB*—genes encoding outer membrane lipoprotein cofactors required for activity, but not expression or stability, of PBP1a and PBP1b, respectively (Typas et al., 2010; Paradis-Bleau et al., 2010; Egan et al., 2014; Lupoli et al., 2014), and 2) point mutations that inactivate PBP1b transpeptidase and/or glycosyltransferase activity but do not impact stability (Meisel et al., 2003).

Implicating PBP activity in growth across pH environments, loss of the cofactors LpoA and LpoB mimicked the pH-dependent growth defects of loss of the enzymes themselves. Analogous to cells defective for PBP1b, deletion of *lpoB* prevented growth at pH 4.8. Likewise, loss of PBP1a’s cofactor LpoA led to a significant defect in DPH between pH values 5.9–8.2 (Figure 2D; Figure 2—figure supplement 2). Interestingly, loss of either Lpo protein did not perfectly recapitulate loss of its cognate class A PBP (Figure 2—figure supplement 2; Figure 2A–C), suggesting the presence of additional relevant regulators in vivo. Complementation analysis of PBP1b variants at acidic pH further bolstered the conclusion that activity is required for pH-dependent growth. As expected, production of wild-type PBP1b in trans restored growth of the ∆*mrcB* mutant at pH 4.8; however, production of PBP1b variants rendering the transpeptidase (S510A, TP*), glycosyltransferase (E233Q, GT*), or both enzymatic activities inactive (TP*GT*) failed to complement growth (Figure 2E). It should be noted that the mutation in the glycosyltransferase active site (E233Q) previously has been shown to attenuate transpeptidase activity by 90%, consistent with observations that PBP transpeptidase activity cannot be assayed in vitro in the absence of functional glycosyltransferase activity (Egan et al., 2014; Bertsche et al., 2005; Terrak et al., 1999; Born et al., 2006). Thus, although our data demonstrate that transpeptidase activity is critical for pH-dependent growth, we cannot discern whether glycosyltransferase activity alone is required.

### Class A PBP activity promotes cell wall integrity across pH environments

Although these findings establish PBP1a and PBP1b activity as essential for optimal fitness across a wide pH range, it remained unclear whether these mutants’ pH-dependent defects in DPH in bulk culture were due to reduction in growth across the population (i.e. decreased rate of mass accumulation and cell expansion) or lysis of a fraction of cells in the population. To differentiate between these two mechanisms, we inoculated early exponential phase (OD_600_ ~0.05–0.1) wild type or mutant cells cultured at pH 6.9 on to agarose pads buffered to pH 4.5 or pH 8.0 and examined cells for incorporation of the dye propidium iodide (PI), which permeates cells with compromised membranes, by microscopy.

**Figure 3.**
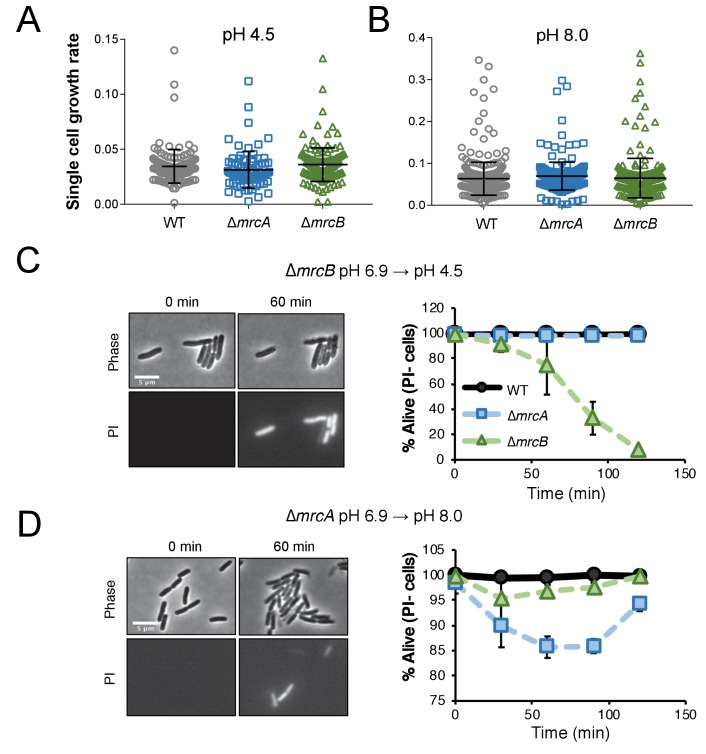
Cells defective for class A PBPs lyse upon exposure to non-permissive pH conditions. (**A–B**) Single cell elongation rates for wild-type (MG1655), ∆*mrcA* (EAM543), and ∆*mrcB* (EAM546) cells during growth on agarose pads at pH 4.5 (A; n = 134, 81, and 155 cells) and pH 8.0 (B; n = 386, 257, and 246 cells). Rates were determined in the MATLAB-based program SuperSegger as described in the methods. Error bars represent SD. (**C–D**) Micrographs depicting representative images of propidium iodide incorporation in ∆*mrcA* (D, left) and ∆*mrcB* (C, left) mutants at t = 0 or 60 min post indicated pH shift. Scale bar represents 5 μm. Cell viability curves for wild-type, ∆*mrcA* (PBP1a), and ∆*mrcB* (PBP1b) strains after acidic (D, right) or alkaline pH (C, right) shift as indicated. Cell death was determined by uniform cytoplasmic staining with propidium iodide. Markers indicate mean percent viability ± SD of three biological replicates. Greater than 100 cells were analyzed for each strain at each time point per replicate.

Consistent with a lytic origin, extensive PI incorporation was observed at one hour post-shift for PBP1b and PBP1a defective cells that underwent acid (pH 6.9 to pH 4.5) or alkaline (pH 6.9 to pH 8.0) shock, respectively (Figure 3C,D). To confirm *bona fide* lysis, we transformed a plasmid expressing *gfp* in to the mutants and measured PI incorporation and loss of cytoplasmic GFP concurrently. Without exception, PI+ cells lacked cytoplasmic GFP signal (Figure 3—figure supplement 1).

**Figure 3—figure supplement 1.**
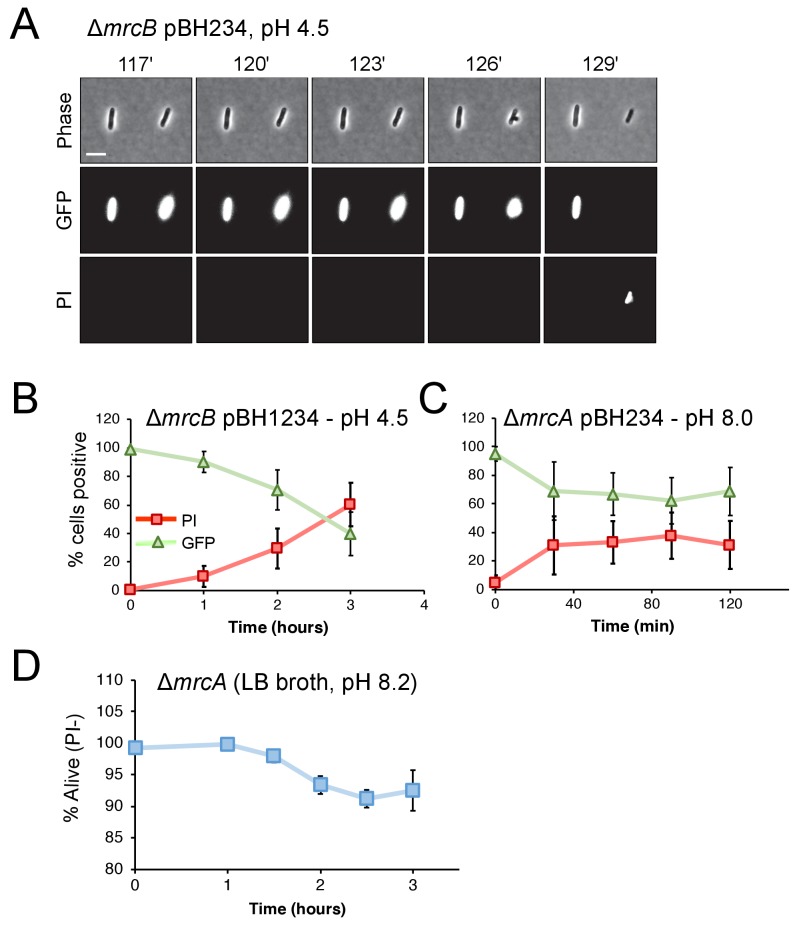
Cytoplasmic GFP loss correlates with propidium iodide staining. Representative micrographs for ∆*mrcB* (EAM546) (**A**) and time course of fluorescence signal (**B–C**) for ∆*mrcA* (EAM543) and ∆*mrcB* mutants expressing *gfp* from an IPTG-inducible promoter (pBH234) when exposed to indicated non-permissive pH conditions. EAM543 and EAM546 were induced with 10 and 20 μM IPTG, respectively. Markers represent percent of cells straining positive for GFP (triangles) or PI (squares) ± SD for three independent biological replicates. (**D**) Propidium iodide staining for ∆*mrcA* (EAM543) cells grown in liquid culture at pH 8.2 at indicated time points. Markers indicate mean percent viability ± SD of three biological replicates. For each time point, at least 100 cells were analyzed per replicate (panels B-D).

Time lapse imaging of cells following pH shift shed light on lysis kinetics: upon pH downshift, ∆*mrcB* cells began to incorporate PI by 30 min (~15% cells labeled), with ~95–100% of cells labeled by two hours post-shift. Negligible cell death was observed for the parental strain or for cells defective for PBP1a during equivalent acid shock (Figure 3C). Conversely, up to 15% of ∆*mrcA* cells underwent lysis an hour following alkaline shift to pH 8.0 (Figure 3D). Although we did observe reduced rates of lysis for the ∆*mrcA* mutant at later time points, this recovery was not recapitulated in liquid culture and thus was not investigated further (Figure 3—figure supplement 1D). ∆*mrcB* and wild type cells exhibited minimal (<5%) or negligible cell death, respectively, in response to alkaline shift on agarose pads. Prior to cell lysis, single cell growth rate of both mutants and the parental strain were similar at each pH condition (Figure 3A,B), indicating lysis is the sole determinant of decreased DPH observed in bulk culture.

**Figure 4.**
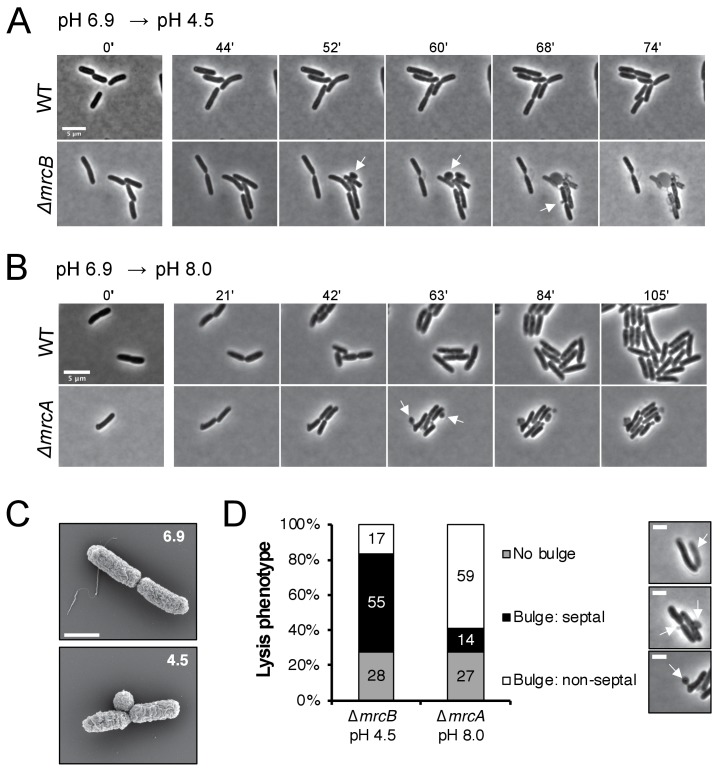
Distinct lytic phenotypes for cells defective for PBP1a and PBP1b upon pH shift. (**A–B**) Representative phase contrast frames of time lapse imaging of ∆*mrcB* (PBP1b; EAM546) and ∆*mrcA* (PBP1a; EAM543) mutants upon acidic (**A**) or alkaline (**B**) pH shift, respectively, as compared to the parental strain (MG1655). White arrows indicate membrane bulges. Scale bar denotes 5 μm. Full videos can be viewed in Figure 4—videos 1 and 2. (**C**) Representative scanning electron microscopy micrographs for ∆*mrcB* (PBP1b; EAM546) mutant shifted to either pH 6.9 or pH 4.5 for two hours prior to fixation. Scale bar represents 1 μm. (**D**) Quantification of lysis phenotype between mutants. Lytic terminal phenotype was categorized into three groups: lysis via septal bulge, non-septal bulge, or no bulge. Determination of lytic phenotype was based on the frames preceding propidium iodide incorporation (time step = 3 min). Micrographs (top to bottom) depict representative images of no bulge, septal bulge, and non-septal bulge, respectively with arrows (scale bar = 2 μm). At least 50 cells across at least two independent biological replicates were assessed (Δ*mrcA*, *n* = 128; Δ*mrcB*, *n* = 278 cells). Bars are subdivided based on percent lytic phenotype in each mutant.

In addition to displaying differential lysis kinetics under their respective non-permissive pH conditions, PBP1a and PBP1b mutants also differed in apparent lysis mechanism. Time lapse imaging during acid shock revealed a high fraction of ∆*mrcB* cells lysed during division, often from a bulge emanating at the septum (Figure 4A, white arrows; Figure 4—video 1). Scanning electron microscopy confirmed the bulges were coincident with the septum in this mutant (Figure 4C). To quantitate the lytic phenotype of the mutants, we categorized the lysis mechanism into three groups: septal bulge, non-septal bulge (including polar and peripheral bulging), and lysis not associated with visible bulging. Septal bulging was determined based on association of the bulge origin with the visible constriction site by phase contrast microscopy. Indeed, this analysis confirmed our observation: 55% of ∆*mrcB* mutants lysed at the septum following pH downshift with the remaining fraction associated with a non-septal bulge (17%) and no bulge (28%) (Figure 4D). In contrast, lysis of the ∆*mrcA* mutant during growth in alkaline pH was not associated with division, and instead, lysis of ~60% of the cells was coincident with a non-septal bulge, typically emanating from the periphery (Figure 4B,D; Figure 4—video 2). These differences may reflect distinct localization preferences of the enzymes (Bertsche et al., 2006; Banzhaf et al., 2012) or disparate weak regions in the PG across pH conditions.

**Figure 4—video 1.** ∆*mrcB* cells undergo septal lysis upon exposure to acidic media. Representative video of ∆*mrcB* cell growth and lysis. Cells were cultured at pH 6.9 to early exponential phase (OD600 ~0.05–0.1) then spotted on agarose pads buffered to pH 4.5. Cells were allowed to dry for 10 min at room temperature prior to imaging at 37°C. Still images corresponding to this video are shown in Figure 4A.

**Figure 4—video 2.** Subpopulation of ∆*mrcA* cells lyse upon exposure to alkaline media. Representative video of ∆*mrcA* cell growth and lysis. Cells were cultured at pH 6.9 to early exponential phase (OD600 ~0.05–0.1) then spotted on agarose pads buffered to pH 8.0. Cells were allowed to dry for 10 min at room temperature prior to imaging at 37°C. Still images corresponding to this video are shown in Figure 4B.

### PBP1a localization and activity are impaired at low pH

Although our data support a model in which class A PBP activity is differentially required for cell wall integrity across pH environments, the mechanistic basis for pH specialization remained unclear. To interrogate this, we compared the production, localization, and biochemical activity of PBP1a and PBP1b as a function of pH.

**Figure 5.**
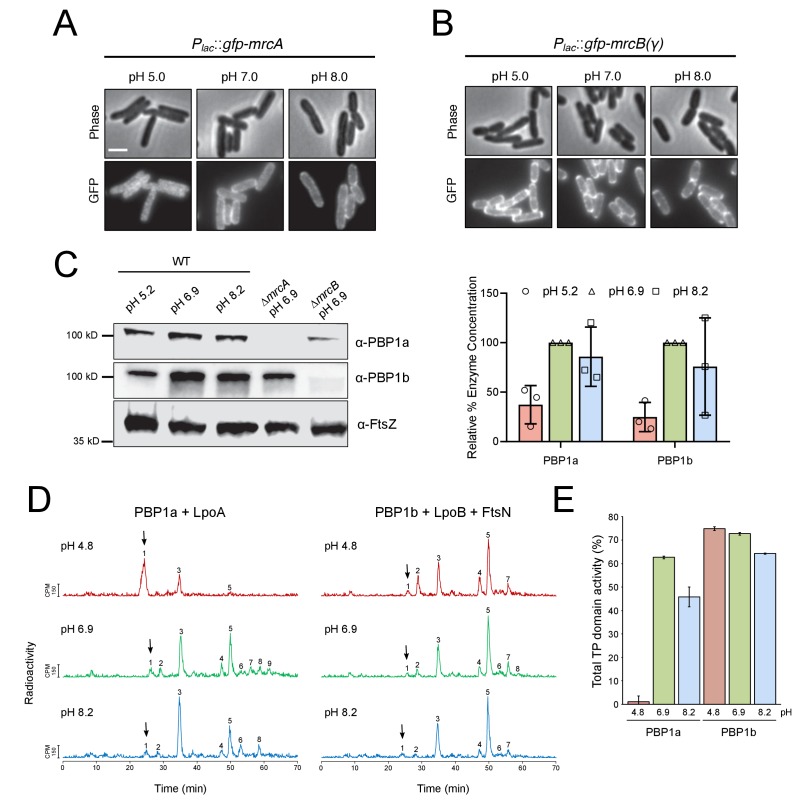
pH-dependence of aPBP localization, production and activity. (**A, B**) Representative micrographs illustrating aPBP localization in strains expressing *P_lac_::gfp-mrcA* (EAM707) and *P_lac_::gfp-mrcB* (EAM718) grown in AB minimal media supplemented with 0.2% maltose and 250 μM IPTG at pH 5.0, 7.0 and 8.0. Scale bar indicates 2 μm. (**C**) Western blot depicting representative biological replicates of PBP1a, PBP1b, and FtsZ levels in wild-type cells (MG1655) cultured at pH 4.8, 6.9, and 8.2. Percent aPBP level (using FtsZ levels as an internal loading control and normalized to pH 6.9 values) across pH conditions is shown to the right. Ponceau staining for total protein levels can be viewed in Figure 5—figure supplement 1. (**D**) Representative HPLC chromatograms of muropeptide analysis. Peak 1 (black arrows), Penta-*P* (stems from remaining lipid II/glycan chain ends); peak 2; Tetra (GT and CPase product); peak 3, Penta (GTase product); peak 4, TetraTetra (GTase, TPase and CPase product); peak 5, TetraPenta (GTase and TPase product), peak 6, TetraTetraTetra (GTase, TPase and CPase product); peak 7, TetraTetraPenta (GTase and TPase product); peak 8, TetraTetraTetraTetra (GTase, TPase and CPase product). (**E**) Quantification of TPase domain activity (sum of TPase and CPase products) of PBP1A + LpoA and PBP1B + LpoB and FtsN at pH 4.8, 6.9 and 8.2. Data is the mean ± range of two replicates. Corresponding representative HPLC chromatograms are shown in D.

We predicted differential PBP production across pH conditions may contribute to the enzymes’ specialization, as has previously been shown for acid-specialist carboxypeptidase PBP6b (Peters et al., 2016b). Consistent with previous proteomic mass spectrometry data (Schmidt et al., 2016), bulk protein levels of both class A PBPs were modestly reduced in acidic media (Figure 5C; Figure 5—figure supplement 1). However, pH-dependent differences in production did not suggest a correlation between either class A PBP’s levels and its contribution to fitness in a particular pH environment.

**Figure 5—figure supplement 1.**
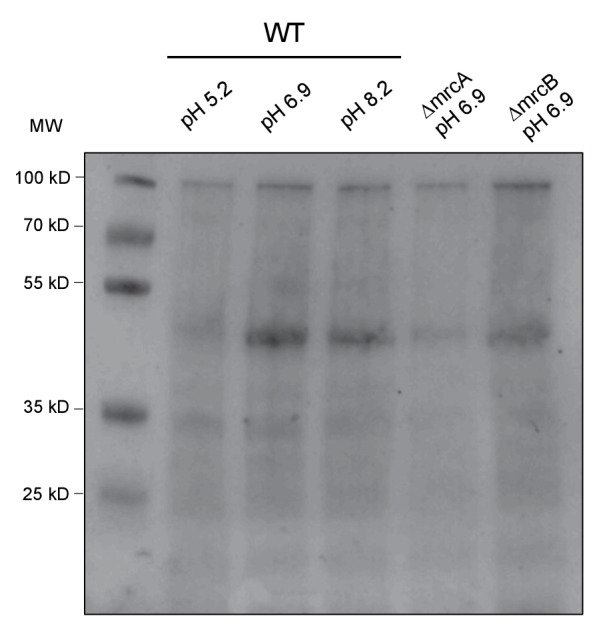
Total protein quantification of class A PBP Western Blot. Corresponding Ponceau stain for blot shown in Figure 5C.

**Figure 5—figure supplement 2.**
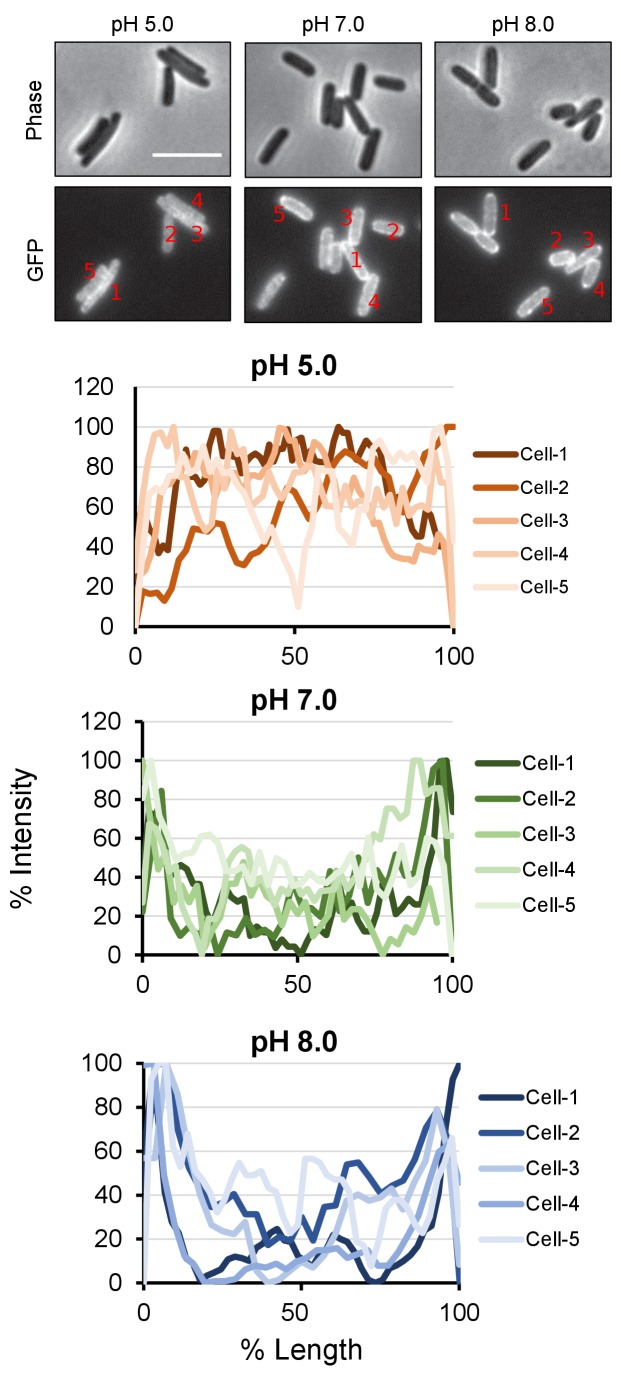
PBP1a peripheral localization across pH conditions. Quantification of class A PBP localization at the cell periphery at pH 5.0, 7.0, and 8.0. For indicated representative cells, a line was drawn across the mid-cell from pole to pole and an intensity profile was generated in FIJI. Cell length and maximum fluorescence intensity were normalized to 100% for each cell.

**Figure 5—figure supplement 3.**
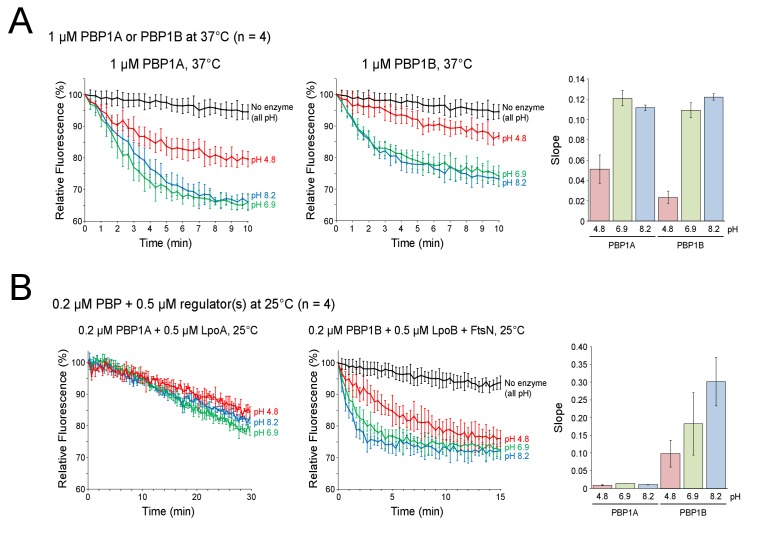
Influence of pH on class A PBP polymerization rate in continuous fluorescence glycosyltransferase assay. Mean relative fluorescence (%), using the start-point as 100%, is plotted against time in minutes (min). The pH of each reaction is shown next to the corresponding curve in the same color, the control sample with no enzyme is shown in black. Polymerization of the fluorescently labelled lipid II causes a decrease in fluorescence signal. Thus, the slope of these plots (shown to the right) gives a relative measure of the GTase rate (n = 4). Data for enzyme alone (**A**) or in combination with indicated activators (**B**) is depicted as mean ± SD for both fluorescence time-point and slope measurements.

**Figure 5—figure supplement 4.**
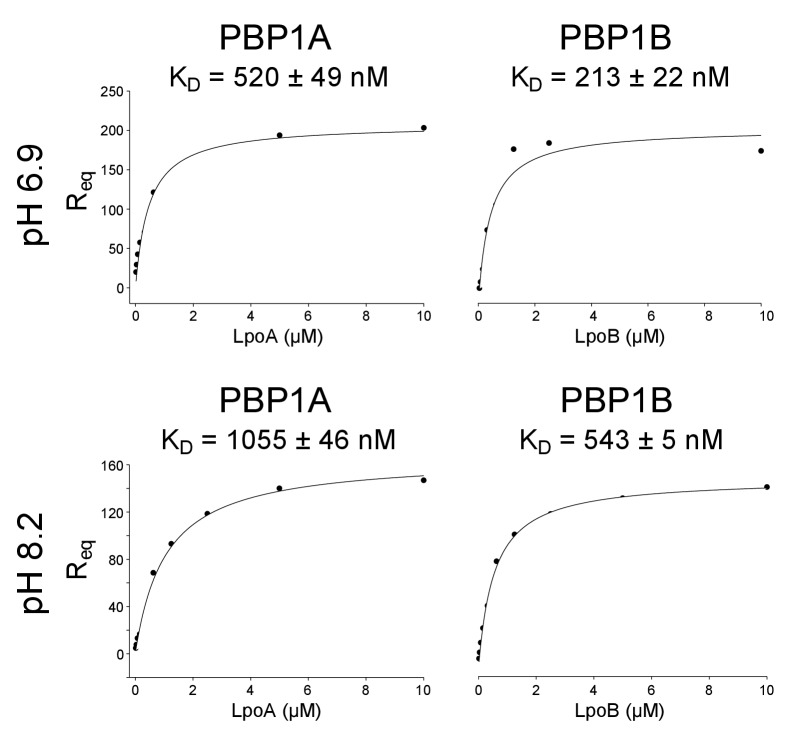
Influence of pH on PBP-lpo binding affinity. Representative binding curves of PBP – Lpo interaction assays at pH 6.9 and 8.2 (indicated to the left). The concentration of Lpo protein injected is plotted against the response its specific binding elicited at equilibrium (R_eq_). Non-linear regression assuming one-site saturation was used to calculate the dissociation constant, K_D_. The K_D_ is the mean ± SD of three replicates.

We next turned to examining PBP localization across pH conditions, using functional GFP fusions to PBP1a and PBP1b produced from the *attHK* locus and under IPTG inducible control (Paradis-Bleau et al., 2010). Similar to previous reports (Bertsche et al., 2006; Paradis-Bleau et al., 2014), the fusion proteins exhibited discrete localization profiles at neutral pH: GFP-PBP1a localized predominantly to the cell periphery, while GFP-PBP1b was present at both the periphery and the septum. Although GFP-PBP1b localization did not noticeably differ across pH conditions, GFP-PBP1a peripheral signal was reduced at pH 5.0, and the fusion adopted an irregular clustered distribution throughout the cell body (Figure 5A,B; Figure 5—figure supplement 2). The physiological significance of this phenotype remains unclear and requires additional investigation.

To examine the effect of pH on PBP synthase activity, we first utilized an end-point assay that concurrently measures glycosyltransferase activity and transpeptidase domain activity, which are coupled in the bifunctional class A PBPs (Bertsche et al., 2005; Born et al., 2006; Egan et al., 2018). Although purified PBP1a and PBP1b exhibit biochemical activity alone in vitro, we chose to test the influence of pH on the class A PBPs in the context of their key cellular activators, including LpoA for PBP1a and LpoB and FtsN for PBP1b. The outer membrane lipoprotein activators LpoA and LpoB are required for the function of their cognate class A PBP in vivo (Typas et al., 2012; Paradis-Bleau et al., 2010), and the essential division protein FtsN can act synergistically with LpoB to enhance PBP1b glycosyltransferase activity up to 16-fold in vitro (Egan et al., 2015).

Briefly, purified enzymes and their cognate activators were solubilized into detergent micelles and incubated with [^14^C]lipid II precursor in buffer at pH 4.8, 6.9, and 8.2. After one hour, the resulting PG was digested into muropeptides and resolved by high performance liquid chromatography. Glycosyltransferase activity is reflected qualitatively in the proportion of [^14^C]lipid II utilized, resulting in a decrease in peak 1. Transpeptidase activity—including both crosslinking activity and carboxypeptidase activity—is quantified by the fraction of muropeptides with modified peptides (peaks 5–9) (Egan et al., 2015). Strikingly, at pH 4.8 PBP1a + LpoA exhibited little glycosyltransferase or transpeptidase domain activity; in this condition the majority of [^14^C]lipid II precursor was not polymerized (Figure 5D, peak 1). In contrast, PBP1b + LpoB + FtsN maintained similar end-point activity across all tested pH conditions (Figure 5D,E). Low PBP1a glycosyltransferase activity at pH 4.8 was confirmed in a continuous fluorescence assay, which measures the polymerization rate of a Dansyl-labeled Lipid II substrate. Consistent with previous work on PBP1b (Egan et al., 2014), in the absence of their cognate activators, both PBP1a and PBP1b exhibited reduced rates of glycosyltransferase activity in acidic conditions. However, co-incubation of PBP1b with LpoB and division protein FtsN significantly increased its polymerization rate under all pH conditions, while PBP1a exhibited poor activity even in the presence of LpoA (Figure 5—figure supplement 3).

To test whether pH-dependent changes in PBP activity reflect a change in affinity of the enzymes for their cognate lipoprotein activators, we performed surface plasmon resonance experiments in which PBP1a and PBP1b were immobilized to chips and exposed to LpoA or LpoB at various concentrations. PBP1a-LpoA and PBP1b-LpoB bound at K_D_ values of 520 ± 49 nM and 213 ± 22 nM at pH 6.9, respectively. Both K_D_ values were ~2 fold higher at pH 8.2 (Figure 5—figure supplement 4), but affinity values could not be determined at pH 4.8 due to significant non-specific binding of both LpoA and LpoB to the chip. Altogether, our data support a model in which PBP1a activity is reduced in acidic conditions, rendering the cell reliant on PBP1b for cell wall integrity and viability.

### Low pH promotes intrinsic resistance to PBP2 and PBP3-specific β-lactams

Combined with the recent findings of other groups (Peters et al., 2016b; Castanheira et al., 2017; Montón Silva et al., 2018), our results suggest that the active cell wall synthesis machinery varies across pH environments. We hypothesized that one potential consequence of environmental plasticity in the cell wall synthesis machinery may be changes in intrinsic resistance to cell wall active antibiotics. If true, condition-dependent intrinsic resistance may have important implications for treatment of *E. coli* infections in host niches with variable pH (Watson et al., 1972; Henderson and Palmer, 1912). To test this model, we measured the minimum inhibitory concentration (MIC) of a panel of compounds during growth of *E. coli* strain MG1655 in a range of physiologically relevant pH conditions (pH 4.5–8.0). We focused on the β-lactam class of antibiotics, which often target specific PBPs at a drug’s MIC (Kocaoglu and Carlson, 2015).

**Figure 6.**
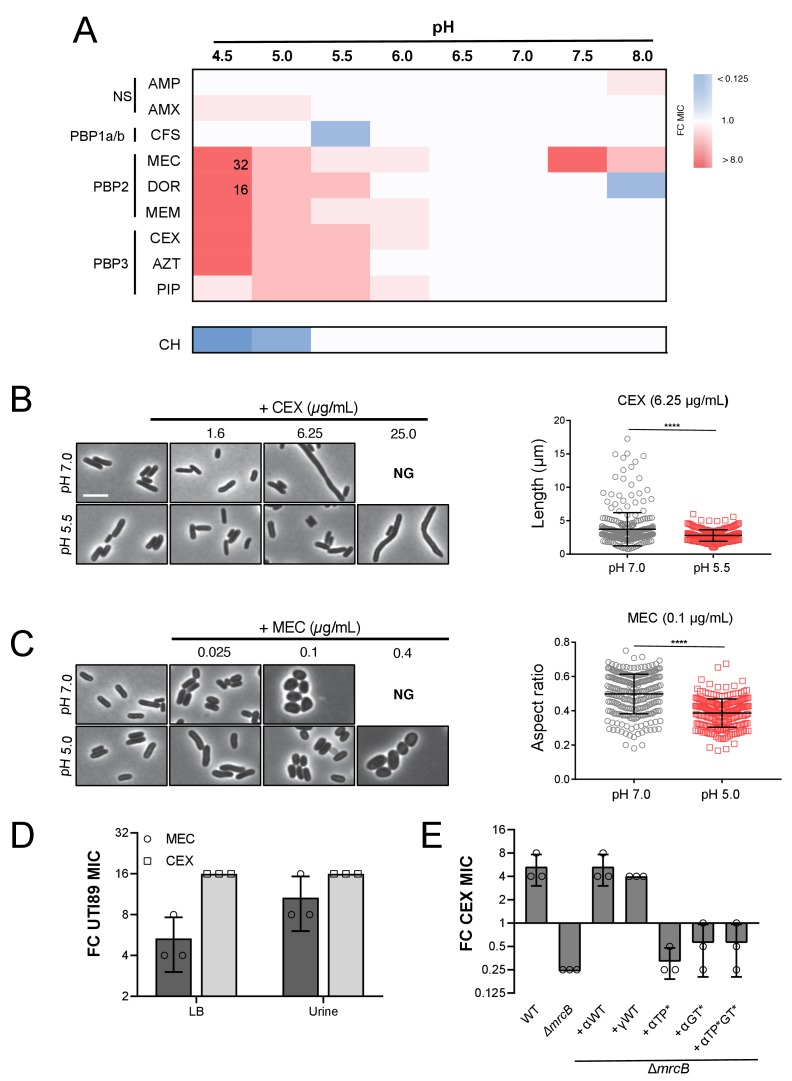
Intrinsic resistance to PBP2 and PBP3-targeting β-lactams at low pH. (**A**) Heat map summarizing fold change in minimum inhibitory concentrations (MIC) of antibiotics for strain MG1655 cultured in LB (pH 4.5–8.0) after 20 hr. Cells in heat map are colored based on median fold change (FC) in MIC at indicated pH compared to pH 7.0 from at least three biological replicates. Fold change values of >8 are indicated in black inside relevant cell. Untransformed median MIC values can be viewed in Supplementary file 4. Abbreviations for antibiotic names are as follows: AMP, ampicillin; AMX, amoxicillin; CFS, cefsulodin; MEC, mecillinam; DOR, doripenem; MEM, meropenem; CEX, cephalexin; AZT, aztreonam; PIP, piperacillin; CH, chloramphenicol. Predominant cellular PBP target is indicated to the left. (**B**) Representative micrographs of cells treated with PBP3 inhibitor cephalexin (CEX) at pH 7.0 and pH 5.5. Distribution of cell lengths at sub-MIC concentration at pH 7.0 and 5.5 (n = 303 and 250) are shown to the right. (**B**) Representative micrographs of cells treated with PBP2 inhibitor mecillinam (MEC) cultured at pH 7.0 or pH 5.0. Distribution of cell aspect ratios (width/length) at sub-MIC concentration at pH 7.0 and 5.0 (n = 250 and 250) are shown to the right. For both panels A and B, scale bar indicates 3 μm, and NG denotes ‘no growth’ observed at the indicated concentration of antibiotic. Error bars represent SD. Significance was assessed by a Kruskal-Wallis test with asterisks denoting significance as follows: ****, p<0.0001. (**D**) Fold change in minimum inhibitory concentration of *E. coli* strain UTI89 to cephalexin (CEX) and mecillinam (MEC) grown at pH 5.0 compared to pH 7.0 in broth culture and in urine. Untransformed MIC values can be viewed in Supplementary file 4. (**E**) Fold change in minimum inhibitory concentration to cephalexin (CEX) for indicated strains grown at pH 5.5 compared to pH 7.0. EAM696 (Δ*mrcB*) derivatives producing PBP1b variants were grown in the presence of 10 μM IPTG. Untransformed MIC values can be viewed in Supplementary file 5. Bars represent mean fold change in minimum inhibitory concentration ± SD across at least three biological replicates.

In support of our hypothesis, we observed a 4 to 32-fold increase in MIC to a subset of the tested β-lactams at pH values < 6.0 (Figure 6A; Supplementary file 4). In particular, cells displayed an increase in intrinsic resistance to compounds that specifically target PBP2 and PBP3, class B PBPs that are essential for cell elongation and division, respectively (Kocaoglu and Carlson, 2015). Consistent with acidic pH conferring a protective effect on the elongation and division machinery and previous reports (Goodell et al., 1976), cells cultured in low pH media retained near-normal morphology in the presence of concentrations of the compounds that led to either filamentation (cephalexin, CEX) or cell rounding (mecillinam, MEC) at pH 7.0 (Figure 6B,C; Figure 6—figure supplement 2). The pH-dependent change in intrinsic resistance was not limited to our laboratory strain: uropathogenic *E. coli* isolate UTI89 (Chen et al., 2006) exhibited a comparable change in MIC to both cephalexin (CEX) and mecillinam (MEC) at low pH during growth in both broth culture and in urine (Figure 6D; Supplementary file 5). In contrast, susceptibility to non-specific β-lactams (ampicillin, AMP; amoxicillin, AMX), a class A PBP-targeting compound (cefsulodin, CFS) (Kocaoglu and Carlson, 2015), or a protein synthesis inhibitor (chloramphenicol, CH) was not strongly pH-dependent. To rule out alternative causes of antibiotic resistance, we confirmed that differences in drug susceptibility could not be attributed to pH-dependent changes in antibiotic stability, proton motive force, β-lactamase production, or outer membrane permeability (Figure 6—figure supplements 1 and 3). These findings suggest that pH-mediated plasticity in the cell wall synthesis machinery influences intrinsic β-lactam sensitivity.

**Figure 6—figure supplement 1.**
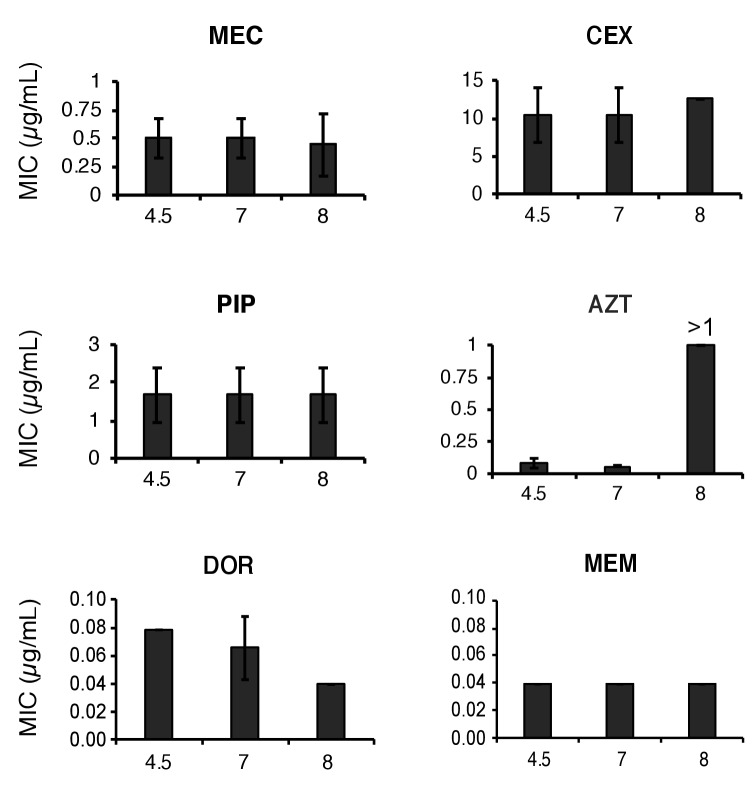
Stability of β-lactam antibiotics across pH values. Mecillinam (MEC), cephalexin (CEX), piperacillin (PIP), aztreonam (AZT), doripenem (DOR) and meropenem (MEM) were incubated in LB media at pH 4.5, 7.0, or 8.0 for 20 hr then inoculated into microtiter dishes with MG1655 for determination of the minimum inhibitory concentration as previously described. Bars represent mean minimum inhibitory concentration ± SD from at least three independent biological replicates. AZT was the only antibiotic to exhibit instability and only at pH 8.0.

**Figure 6—figure supplement 2.**
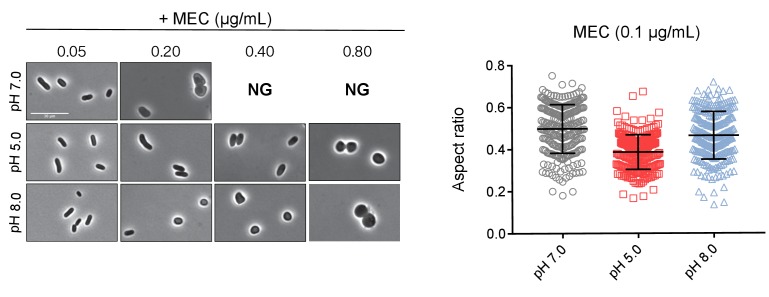
Mecillinam resistant cells at pH 8.0 remain rounded. Representative micrographs and aspect ratio (width/length) quantification of cells treated with PBP2 inhibitor mecillinam for twenty hours at pH 5.0, 7.0, and 8.0 (n = 250 for each group). Error bars indicate SD, and scale bar represents 10 μm.

**Figure 6—figure supplement 3.**
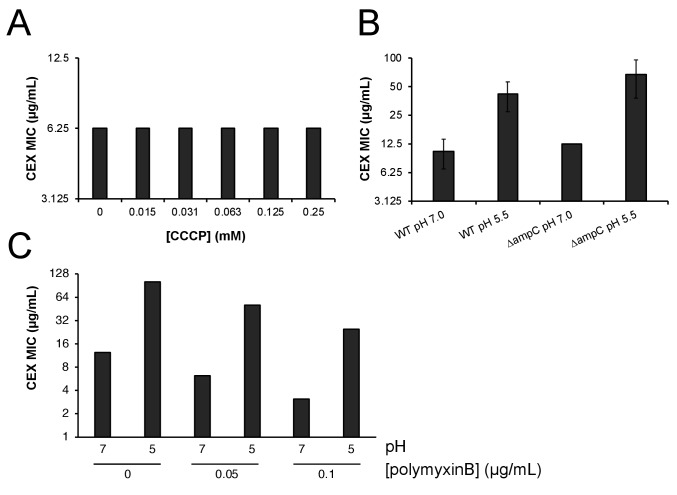
Proton motive force, AmpC β-lactamase, and outer membrane permeability do not confer pH-dependent resistance to cephalexin. (**A**) Minimum inhibitory concentration of cephalexin at pH 7.0 in the presence of various concentrations of proton motive force inhibitor carbonyl cyanide-m-chlorophenylhydrazone (CCCP). (**B**) Comparison of the minimum inhibitory concentration of cephalexin at pH 7.0 and pH 5.5 between wild type (MG1655) and Δ*ampC* (EAM749) cells. Markers denote mean minimum inhibitory concentration ± SD deviation from three biological replicates. (**C**) Comparison of the minimum inhibitory concentration of cephalexin at pH 7.0 and pH 5.0 in wild type (MG1655) in the presence or absence of sub-growth inhibitory concentrations of pore-forming antibiotic polymyxin B.

**Figure 6—figure supplement 4.**
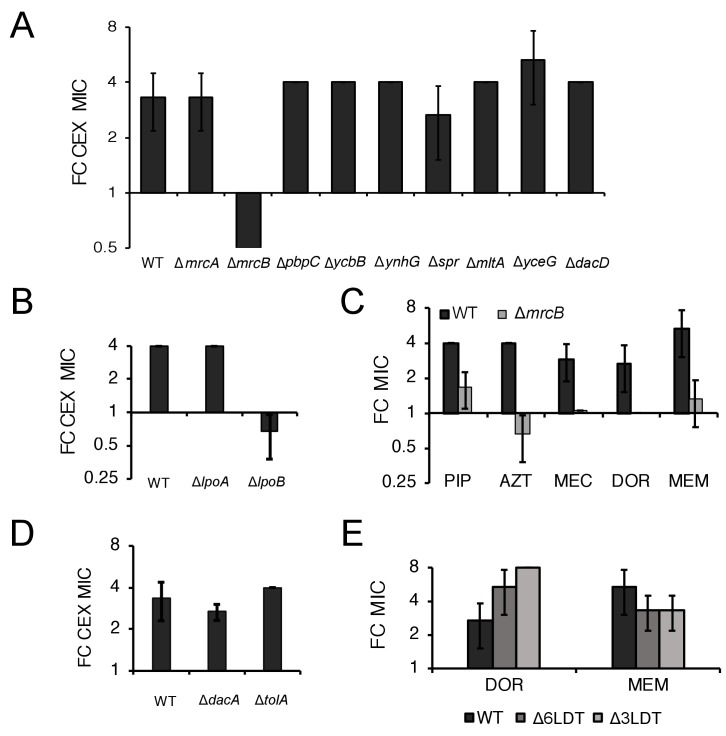
Δ*mrcB* abolishes low pH-dependent resistance independent of growth rate and β-lactam sensitivity. Comparison in the fold change in cephalexin (CEX) minimum inhibitory concentration of mutants in genes encoding nonessential transpeptidases and pH specialist autolysins (**A**) or lipoprotein activators (**B**) at pH 5.5 compared to pH 7.0. (**C**) Comparison of the fold change in minimum inhibitory concentration of piperacillin (PIP), aztreonam (AZT), mecillinam (MEC), doripenem (DOR), and meropenem (MEM) for wild type and Δ*mrcB* mutant cells at pH 5.0 compared to pH 7.0. (**D**) Comparison of the fold change in minimum inhibitory concentration of cephalexin (CEX) across wild type, Δ*dacA*, and Δ*tolA* cells at pH 5.5 compared to pH 7.0. (**E**) Comparison of the fold change in minimum inhibitory concentration of doripenem (DOR) and meropenem (MEM) in cells lacking three (EAM1152) or six LD-transpeptidases (BW25113∆6LDT) at pH 5.0 compared to pH 7.0. In all panels, bars represent mean minimum inhibitory concentration ± SD across three independent biological replicates.

### PBP1b is required for low pH-dependent β-lactam resistance

We next sought to identify which, if any, cell wall enzymes were required for resistance to PBP2 and PBP3-targeting compounds in acidic media. We narrowed our focus to three classes of enzymes: 1) non-essential transpeptidases, including the class A PBPs and the LD-transpeptidases, which have been implicated in β-lactam resistance (Lai et al., 2017; Hugonnet et al., 2016; Peters et al., 2018); 2) pH specialist autolysins identified in this work; and 3) PBP6b, a carboxypeptidase required for proper morphology—but not growth—in acidic media (Peters et al., 2016b). Mutants defective for production of each enzyme were tested for loss of resistance to CEX at pH 5.5 compared to pH 7.0.

Genetic analysis suggests that PBP1b activity is specifically required for pH-dependent resistance to β-lactam antibiotics. Strains defective for production of PBP1a, PBP1c, LdtD, LdtE, MepS, MltA, MltG, and PBP6b exhibited a similar increase in resistance to CEX at pH 5.5 as the parental strain (Figure 6—figure supplement 4A). In contrast, loss of PBP1b abolished resistance at pH 5.5 and in fact, slightly increased susceptibility to CEX (Figure 6E). This phenotype was not specific to CEX: resistance to other PBP2 and PBP3 targeting compounds was also eliminated or significantly reduced in cells defective for PBP1b (Figure 6—figure supplement 4C). PBP1b enzymatic activity was required for resistance. Loss of the enzyme’s cognate outer membrane lipoprotein LpoB or inactivation of its catalytic activity eliminated CEX resistance at low pH (Figure 6E; Figure 6—figure supplement 3B; Supplementary file 6). Importantly, a mutant with a comparable growth rate defect at pH 5.5 (Δ*mrcB* 1.91 ± 0.02 DPH; Δ*tolA* 1.40 ± 0.04 DPH) still displayed the same fold change in resistance to CEX at pH 5.5 as the parental strain. Likewise, a mutant in PBP5 (Δ*dacA)* with increased sensitivity to CEX even at neutral pH, similar to Δ*mrcB* (Schmidt et al., 1981; García del Portillo and de Pedro, 1990), also retained the resistance phenotype in acidic growth conditions (Sarkar et al., 2010) (Figure 6—figure supplement 3D). In sum, our findings point to a specific role for PBP1b in intrinsic β-lactam resistance in acidic media.

## Discussion

### Class A PBPs protect cell wall integrity across environmental conditions

By varying a single growth parameter—extracellular pH—we uncovered specialized roles for a subset of nonessential cell wall synthases and autolysins in *E. coli* that previously had been classified as redundant for growth. Of the pH specialist enzymes identified, we focused on the bifunctional synthases PBP1a and PBP1b, which we find are required for cell wall integrity in distinct pH environments. Failure to produce PBP1b in acidic media (pH <5.9) or PBP1a in more alkaline conditions (pH 6.5–8.2) reduced fitness and led to cell lysis (Figure 2 and Figure 3). This lytic death is characteristic of class A PBP-deficient cells (Suzuki et al., 1978) and is consistent with their essential role in PG synthesis, perhaps by filling gaps in the PG foundation (Cho et al., 2016). Importantly, a recent study failed to observe major differences in global PG composition in *E. coli* cells grown in pH 7.5 and pH 5.0 (Peters et al., 2016b). Hence, the differential requirement for the class A PBPs in growth across pH conditions is unlikely to be a consequence of altered cell wall structure or distinct enzymatic activity of the PBPs.

Instead, our data suggest that pH-dependent differences in class A PBP activity underlie their contribution to maximal fitness in distinct pH environments. Even in the presence of its activator LpoA, PBP1a exhibits little glycosyltransferase and transpeptidase activity in vitro in acidic conditions, rendering the cell reliant on PBP1b to provide the essential class A PBP activity (Figure 5D,E; Figure 5—figure supplement 3). At present, the precise mechanism for reduced activity of PBP1a in acidic media remains unclear; possible causes include pH-dependent changes in structure, substrate binding, or catalysis, as well as reduced affinity to LpoA in acidic conditions. We attempted to test the latter model using surface plasmon resonance (SPR), but technical limitations prevented us from drawing firm conclusions (Figure 5—figure supplement 4). Although further work is needed, the depletion of PBP1a from the cell periphery in acidic media may also reflect an inactive state of the protein in vivo (Figure 5A).

Unlike PBP1a, PBP1b activity is largely invariant in vitro across the pH conditions tested when assayed in the presence of its key regulators (Figure 5D,E; Figure 5—figure supplement 3). Although this finding is consistent with our observation that PBP1b can partially compensate for loss of PBP1a in alkaline media (Figure 2), it remains unclear why PBP1a is required for maximal fitness in alkaline media. Additional factors in vivo, such as novel class A PBP regulators, may be responsible for the cell’s preference for PBP1a in alkaline conditions that are not recapitulated in our in vitro assays. In support of this model, PBP1b exhibits reduced binding to radiolabeled penicillin in membrane extracts incubated in alkaline buffer relative to neutral or acidic conditions (Amaral et al., 1986). Alternatively, differences in the enzymes’ localization and/or interaction partners may also play a role in their pH specialization. For example, PBP1a and PBP1b preferentially associate with specialized cell wall synthesis complexes essential for cell elongation and cell division, respectively (Müller et al., 2007; Bertsche et al., 2006; Banzhaf et al., 2012). Our current study was limited to interrogating the pH specialization of nonessential cell wall enzymes. Future work investigating the influence of pH on the essential components of the elongation and division machinery is thus necessary to determine whether the activity of these complexes affects the cell’s class A PBP preference.

Although PBP1a and PBP1b share an essential role in growth and cell wall integrity under standard culture conditions (i.e. nutrient rich, neutral pH growth medium) (Suzuki et al., 1978), there had been previous hints these enzymes were not interchangeable. As previously mentioned, each class A PBP possess unique interaction partners and subcellular localization profiles (Müller et al., 2007; Bertsche et al., 2006; Banzhaf et al., 2012; Gray et al., 2015; Leclercq et al., 2017). Mutants defective for each enzyme also display differential susceptibility to antibiotic treatment (discussed below), osmotic shifts, and mechanical stress (Yousif et al., 1985; Paradis-Bleau et al., 2014; Auer et al., 2016) and have different roles in de novo regeneration of rod shape (Ranjit et al., 2017). We anticipate further study of these synthases—as with other ‘redundant’ cell wall autolysins—under nonstandard culture conditions will continue to reveal unique roles for these enzymes in cell wall biogenesis.

### Plasticity in cell wall metabolism influences intrinsic resistance to cell wall active antibiotics

Analogous to alternative PBP usage in methicillin resistant *Staphylococcus aureus* (Berger-Bächi, 1999; Chan et al., 2016), our results suggest that environment-driven plasticity in *E. coli* PG synthesis may have consequences for intrinsic resistance to β-lactam antibiotics with a narrow target specificity (Figure 6). Strikingly, we find that culturing *E. coli* in low pH media is sufficient increase intrinsic resistance to PBP2 and PBP3-targeting β-lactams up to 32-fold. Considering *E. coli* encounters a wide range of pH environments across host niches (Watson et al., 1972; Bilobrov et al., 1990), our observation reinforces the importance of conducting antibiotic susceptibility testing under physiologically relevant conditions (Ersoy et al., 2017; Thulin et al., 2017).

Mechanistically, our data demonstrate low pH-dependent resistance specifically requires PBP1b activity. PBP1b has previously been implicated in intrinsic resistance to β-lactam antibiotics: cells defective for PBP1b or LpoB are hypersensitive to a wide variety of β-lactams (Nichols et al., 2011; Paradis-Bleau et al., 2014; García del Portillo and de Pedro, 1990). Likewise, elevated PBP1b activity protects cells from the lethality of the PBP2 specific antibiotic mecillinam (Lai et al., 2017). At present, the precise role for PBP1b in β-lactam protection remains unclear. It appears to be a function specifically endowed to PBP1b, for cells defective for PBP1a production do not display differential β-lactam susceptibility (Nichols et al., 2011; García del Portillo and de Pedro, 1990), highlighting another possible source of specialization between the class A PBPs. Interestingly, we and others have observed cells cultured in acidic media exhibit near normal morphology in the presence of concentrations of PBP2 or PBP3 inhibitors that lead to cell rounding or filamentation at neutral pH (Figure 6B,C) (Goodell et al., 1976). We thus speculate that PBP1b may play a role in preserving the normal functions of the elongation and division machinery in low pH environments. PBP1b may substitute for the essential function of PBP2 and PBP3 in these complexes (Modell et al., 2014) or may indirectly support growth by serving a quality control function (Morè et al., 2019). Clarifying PBP1b’s role in intrinsic resistance to β-lactams will shed light on the mechanics of cell wall biogenesis and inform the design and use of novel antibiotic therapies.

### Apparent redundancy ensures fitness across environmental conditions

Apart from influencing antibiotic resistance, environmental specialization of cell wall enzymes is likely a key adaptation that allows *E. coli* to thrive across an unusually wide pH range (pH ~4–9) and even tolerate extreme pH shocks, such as during transient exposure to gastric acid (pH ~2) (Jordan et al., 1999). Plasticity in the cell wall synthesis machinery likely works in concert with the organism’s ability to modify extracellular pH through the export of acidic and alkaline substrates (Lu et al., 2013; Krulwich et al., 2011). In this context, pH-specialist cell wall enzymes may function in part to maintain cell wall integrity until the extracellular media reaches a growth-permissive pH.

Among other pH tolerant organisms, strategies employed to expand growth across wide pH ranges are likely to vary, even between closely related species. *S. enterica* serovar Typhimurium encodes a PBP3 paralog, termed PBP3_SAL_, that is active during growth in acidic environments, including intracellularly in the phagolysosome (Castanheira et al., 2017). As PBP3_SAL_ is restricted to *Salmonella*, *Enterobacter*, and *Citrobacter* spp., alternative mechanisms to cope with changing pH environments must exist. Elucidating the requirements for pH-dependent growth in organisms outside the *Enterobacteriaceae* will shed light on whether class A PBPs, which are broadly conserved across bacteria (Typas et al., 2010), play a central role in the process.

Apart from pH, we anticipate enzyme specialists exist across environmental conditions; redundancy in cell wall enzymes is present throughout bacterial species, even among those that only grow at a narrow pH range (Pazos et al., 2017). *B. subtilis*, for example, encodes 16 PBPs, yet its growth is restricted to pH 6.0–9.0 (Wilks et al., 2009). Ionic strength, osmolality, and temperature also vary across the diverse habitats bacteria occupy. Like pH, these factors may have significant impacts on the chemical and physical properties of the periplasm and the extracytoplasmic space of Gram-positive bacteria. In support of this idea, PBP2 from *Caulobacter crescentus* displays differential localization patterns as a function of extracellular osmolality (Hocking et al., 2012), and the lytic transglycosylase MltA from *E. coli* is ~10 times more active at 30°C than at 37°C in vitro (van Straaten et al., 2005; Lommatzsch et al., 1997). We expect environmental specialization may also underlie the high levels of redundancy in other periplasmic protein classes, including sugar transporters, efflux pump adapter proteins, and chaperones (Jensen et al., 2002; Smith and Blair, 2014; Rizzitello et al., 2001). Nevertheless, it is clear that improved understanding of the contribution of many enzymes to bacterial fitness in nature demands a departure from standard growth conditions used to study bacterial physiology in the lab.

## Materials and methods

**Key resources table:** 

Reagent type (species) or resource	Designation	Source or reference	Identifiers	Additional information
Gene (*Escherichia coli*)	*mrcB*	NA	EcoCyc:EG10605	
Gene (*E. coli*)	*mrcA*	NA	EcoCyc:EG10748	
Gene (*E. coli*)	*lpoA*	NA	EcoCyc:G7642	
Gene (*E. coli*)	*lpoB*	NA	EcoCyc:G6565	
Strain, strain background (*E. coli*)	MG1655; wild-type	PMID:6271456		
Strain, strain background (*E. coli*)	UTI89	PMID:11402001		
Antibody	anti-PBP1a	Gift of Waldemar Vollmer		(1:5000)
Antibody	anti-PBP1b	Gift of Waldemar Vollmer		(1:1000)
Antibody	anti-FtsZ	Gift of David Weiss		(1:5000)
Recombinant DNA reagent	*mrcB::kan; ∆mrcB*	PMID:16738554; Coli Genetic Stock Center	CGSC:JW0145-1	
Recombinant DNA reagent	*mrcA::kan; ∆mrcA*	PMID:16738554; Coli Genetic Stock Center	CGSC:JW3359-1	
Recombinant DNA reagent	*lpoA::kan; ∆lpoA*	PMID:16738554; Coli Genetic Stock Center	CGSC:JW3116-1	
Recombinant DNA reagent	*lpoB::kan; ∆lpoB*	PMID:16738554; Coli Genetic Stock Center	CGSC:JW5157-1	
Recombinant DNA reagent	pCP20	PMID:10829079		
Recombinant DNA reagent	pUM1Bα	PMID:12949085		
Recombinant DNA reagent	pUM1B*γ*	PMID:12949085		
Recombinant DNA reagent	pUM1Bα*	PMID:12949085		
Recombinant DNA reagent	pUM1BTG*α	PMID:12949085		
Recombinant DNA reagent	pUM1BTG*α*	PMID:12949085		
Chemical compound, drug	Ampicillin Sodium Salt	Sigma Aldrich	Catalog:A9518	
Chemical compound, drug	Amoxicillin	Sigma Aldrich	Catalog:A8523	
Chemical compound, drug	Cefsulodin Sodium Salt Hydrate	Sigma Aldrich	Catalog:C8145	
Chemical compound, drug	Mecillinam	Sigma Aldrich	Catalog:33447	
Chemical compound, drug	Doripenem Hydrate	Sigma Aldrich	Catalog:SML1220	
Chemical compound, drug	Meropenem Hydrate	Sigma Aldrich	Catalog:M2574	
Chemical compound, drug	Cephalexin	Sigma Aldrich	Catalog:33989	
Chemical compound, drug	Aztreonam	Sigma Aldrich	Catalog:A6848	
Chemical compound, drug	Piperacillin Sodium Salt	Sigma Aldrich	Catalog:P8396	
Software, algorithm	SuperSegger	PMID:27569113		
Software, algorithm	FIJI	PMID:22743772		
Other	Propidium iodide	Sigma Aldrich	Catalog:81845	

### Bacterial strains, plasmids, and growth conditions

Unless otherwise indicated, all chemicals, media components, and antibiotics were purchased from Sigma Aldrich (St. Louis, MO). Bacterial strains and plasmids used in this study are listed in Supplementary file 1 and Supplementary file 2, respectively. All deletion alleles were originally provided by the Coli Genetic Stock (Baba et al., 2006) and transduced into *E. coli* strain MG1655. For the hits identified in the growth rate screen, the expected mutation was confirmed by diagnostic PCR with *Taq* polymerase. Unless otherwise indicated, strains were grown in lysogeny broth (LB) media (1% tryptone, 1% NaCl, 0.5% yeast extract) supplemented with 1:10 MMT buffer (1:2:2 molar ratio of D,L-malic acid, MES, and Tris base) to vary media pH values between pH 4–9. AB defined media (Clark and Maaløe, 1967) was fixed to indicated pH values with addition of 5M HCl or 5M NaOH. Uropathogenic *E. coli* strain UTI89 was cultured in urine provided by a healthy donor and supplemented with MMT buffer to fix the pH. When selection was necessary, cultures were supplemented with 50 µg/mL kanamycin (Kan) and 25–100 µg/mL ampicillin (Amp). Cells were grown at 37°C either in 96-well microtiter plates shaking at 567 cpm or in glass culture tubes shaking at 200 rpm for aeration.

### Growth rate measurements

Strains were grown from single colonies in glass culture tubes in LB +MMT buffer (pH 6.9) to mid-log phase (OD_600_ ~0.2–0.6), pelleted, and re-suspended to an OD_600_ of 1.0 (~1×10^9^ CFU/mL). Cells were diluted and inoculated into fresh LB +MMT buffer at various pH values in 96-well plates (150 µl final volume) at 1 × 10^3^ CFU/mL. Uncovered plates sealed with gas permeable membrane strips were grown at 37°C shaking for 20 hr in a BioTek Eon microtiter plate reader, measuring the OD_600_ of each well every ten minutes. Mass doublings per hour (DPH) was calculated by least squares fitting of early exponential growth (OD_600 _0.005–0.1) in R. Best fit lines with an R^2^ value below 0.95 were excluded from further analysis. Examples of growth curves and fit lines are presented in Figure 1—figure supplement 1, along with a sample script (Supplementary file 7) and representative source data (Figure 1—source datas 1–3). To allow for direct comparison between wild type and mutant growth, some panels present % wild type growth, which represents the DPH_Mutant_/DPH_WT_ × 100.

### Microscopy and time lapse imaging

For time lapse imaging experiments, cells were grown from a single colony in LB +MMT buffer (pH 6.9) to early exponential phase (OD_600_ ~0.05–0.1) then mounted onto 1.0% agarose pads at pH 4.5, 6.9, or 8.0. Where indicated, propidium iodide was added to the agarose pad at a final concentration of 1.5 µM. Cells were allowed to dry on pads 10 min prior to imaging. All phase contrast and fluorescence images were acquired on a Nikon Ti-E inverted microscope (Nikon Instruments, Inc) equipped with a 100X Plan N (N.A. = 1.45) Ph3 objective, X-Cite 120 LED light source (Lumen Dynamics), and an OrcaERG CCD camera (Hammamatsu Photonics, Bridgewater, N.J.). Filter sets were purchased from Chroma Technology Corporation. The objective was pre-heated to 37°C using an objective heater. Image capture and analysis was performed using Nikon Elements Advanced Research software. Cell death quantification was determined by cells uniformly stained with propidium iodide, and terminal lytic phenotype of cells was determined by assessment of the frames immediately preceding propidium iodide incorporation. Single cell elongation rate, defined as k = ΔL/Δt/L, was determined in the MATLAB-based program SuperSegger (Stylianidou et al., 2016). Cells that lysed during the movie, indicated by negative k values, were filtered out prior to analysis.

For class A PBP localization studies, cells were grown in AB minimal media supplemented with 0.2% maltose and 250 µM IPTG overnight then sub-cultured into fresh media the following morning. Cells were grown to OD_600 _0.1–0.2 at 37°C then fixed by adding 20 μL of 1M NaPO4, pH 7.4, and 100 μL of fixative (fixative = 1 mL 16% paraformaldehyde + 6.25 μL 8% glutaraldehyde). Samples were incubated at room temperature for 15 min, then on ice for 30 min. Fixed cells were pelleted, washed three times in 1 mL 1X PBS, pH 7.4, then resuspended in GTE buffer (glucose-tris-EDTA) and stored at 4°C. Quantification shown in Figure 5—figure supplement 2 was performed in FIJI (Schindelin et al., 2012). Briefly, an intensity profile was generated for each cell by drawing a line across the midline of the cell from pole to pole. Maximum length and intensity were normalized to 100%. Cells enriched for class A PBP localization at the cell periphery would be expected to have increased intensity at the cell poles (0% and 100% of cell length).

### Scanning electron microscopy

Wild type and Δ*mrcB* cells were grown to mid-exponential phase in MMT buffered pH 6.9 LB media and back-diluted to an OD_600_ = 0.1 into either pH 6.9 or 4.5 media. Cells were allowed to grow for an additional hour, fixed as described above, and applied to poly-lysine coated coverslips. Post fixation, samples were rinsed in PBS 3 times for 10 min each followed by a secondary fixation in 1% OsO_4_ in PBS for 60 min in the dark. The coverslips were then rinsed three times in ultrapure water for 10 min each and dehydrated in a graded ethanol series (50%, 70%, 90%, 100% x2) for 10 min each step. Once dehydrated, coverslips were then loaded into a critical point drier (Leica EM CPD 300, Vienna, Austria) which was set to perform 12 CO_2_ exchanges at the slowest speed. Once dried, coverslips were then mounted on aluminum stubs with carbon adhesive tabs and sputter coated with 6 nm of iridium (Leica ACE 600, Vienna, Austria). After coating, the samples were then loaded into a FE-SEM (Zeiss Merlin, Oberkochen, Germany) imaged at 3 KeV with a probe current of 178 pA using the Everhart Thornley secondary electron detector.

### SDS-PAGE and immunoblotting

Strains were grown from a single colony in LB + 1:10 MMT at pH 4.8, 6.9, or 8.2 to mid-log phase (OD_600_ ~0.2–0.6), back-diluted to 0.005 in 5 mL of media, and grown to an OD_600_ between 0.2–0.3. Samples were pelleted, re-suspended in 2x Laemmli buffer to an OD_600_ ~20, and boiled for ten minutes. Samples (10 µl) were separated on 12% SDS-PAGE gels by standard electrophoresis and transferred to nitrocellulose membranes. Blots were probed with PBP1b (1:1000), PBP1a (1:5000), and FtsZ rabbit antisera (1:5000) and HRP-conjugated secondary antibody (1:2000-1:10000; goat α-rabbit). Blots were imaged on a LiCor Odyssey imager. Quantitation was determined in ImageJ and normalized to FtsZ levels as an internal loading control.

### In vitro protein materials and interaction and activity assays

Lipid II versions were prepared as previously described (Bertsche et al., 2005; Breukink et al., 2003). The following proteins were prepared as previously described; PBP1B (Bertsche et al., 2006), LpoB (Egan et al., 2014), PBP1A (Born et al., 2006), and LpoA (Jean et al., 2014). Antisera against PBP1A and PBP1B were obtained from Eurogentec (Liege, Belgium) and purified over an antigen column as described previously (Bertsche et al., 2006).

SPR experiments were performed as previously described (Egan et al., 2014). LpoA and LpoB samples were prepared for injection over the PBP surface by 1:1 serial dilution from 10 μM to 19.5 nM. Assays were performed in triplicate at 25 ˚C, at a flow rate of 75 μL/min and with an injection time of 5 min. The running buffers consisted of 20 mM of either; sodium acetate pH 4.8, HEPES/NaOH pH 6.9, or Tris/HCl pH 8.2 with 150 mM NaCl, and 0.05% Triton X-100. The dissociation constant (K_D_) was calculated by non-linear regression using SigmaPlot 13 software (Systat Software Inc). Continuous fluorescence GTase assays were performed as described previously (Egan and Vollmer, 2016) with modification. Final buffer composition was 20 mM of either; sodium acetate pH 4.8, HEPES/NaOH pH 6.9, or Tris/HCl pH 8.2 plus 150 mM NaCl, 10 mM MgCl_2_, and 0.05% Triton X-100. Enzymes were assayed alone at 1 μM at 37°C, and at 0.2 μM in the presence of 0.5 μM regulator(s) at 25°C. The muramidase usually included in the assay samples to digest newly synthesized glycans, thereby improving fluorescence signal, was omitted to avoid indirect pH effects on observations. The slopes of the resulting curves correlate with the GTase rate and were calculated at their fastest point using linear regression in Excel 2016 (Microsoft). When presented in Figure 5—figure supplement 3, the values are inverted from negative for simplicity. Measurement of total PG synthesis activity using radiolabelled lipid II substrate was performed as previously described (Biboy et al., 2013) using enzyme (0.5 μM) and regulator(s) (2 μM) in the same three buffers indicated for the GTase assay. Total TPase activity was calculated as the percentage of muropeptide products known to be produced by this domain’s function, including peptide cross-linking and DD-carboxypeptidase activity.

### Antibiotic susceptibility testing

For determination of minimum inhibitory concentrations, cells were grown from a single colony in LB media at the indicated pH to mid-exponential phase (OD_600_ ~0.2–0.6) at 37°C with aeration and then inoculated at 1 × 10^5^ CFU/mL into LB media of the same pH in sterile 96-well plates with a range of two-fold dilutions of the indicated antimicrobial agent (final volume, 150 µL). Plates were incubated at 37°C shaking for 20 hr before determination of the well with the lowest concentration of the antibiotic that had prevented growth by visual inspection.

### Antibiotic stability testing

Antibiotics were incubated for 20 hr in LB media at pH 4.5, 7.0, or 8.0 and then diluted into 96-well plates containing LB media (pH 7) and 1 × 10^5^ CFU/mL MG1655. Plates were then incubated at 37°C shaking for 20 hr before determination of the compound’s minimum inhibitory concentration.

### Terminal phenotype assessment

Cells from minimum inhibitory concentration assays were spotted (5 µL) onto 1.0% agarose pads 20 hr post-treatment and imaged by phase contrast microscopy to track cell morphology in response to antibiotic treatment across pH values. Growth rate was monitored by OD_600_ in the BioTek Eon plate reader to confirm all cells examined were in the same growth phase and at approximately the same optical density prior to imaging. Cell dimensions were quantified in the MATLAB-based program SuperSegger (Stylianidou et al., 2016).

### Quantification and statistical analysis

A minimum of three biological replicates were performed for each experimental condition unless otherwise indicated. Data are expressed as means ± standard deviation (SD) or standard error of the mean. Statistical tests employed are indicated in the text and corresponding figure legend. Analysis was performed in R or GraphPad Prism. Asterisks indicate significance as follows: *, p<0.05; **, p<0.01; ***, p<0.001; ****, p<0.0001.

## Data Availability

All data generated or analyzed during this study are included in the manuscript and supporting files.

## Additional files

Supplementary file 1. Bacterial strains used in this study.

Supplementary file 2. Plasmids used in this study.

Supplementary file 3. Summary of growth rate screen. Supports Figure 1. Presents mean mass doubling time ± standard deviation of each cell wall mutant at pH 4.8, 6.9, and 8.2 during preliminary screen (n = 3).

Supplementary file 4. β-lactam sensitivity of MG1655 across pH conditions. Supports Figure 6A. Presents median minimum inhibitory concentrations of indicated β-lactam antibiotics to MG1655 across pH conditions of at least three biological replicates. Values are represented as μg/mL.

Supplementary file 5. β-lactam sensitivity of UTI89 across pH conditions. Supports Figure 6D. Presents median minimum inhibitory concentrations of cephalexin (CEX) and mecillinam (MEC) to UTI89 across pH conditions in LB and in urine (n = 3). Values are represented as μg/mL.

Supplementary file 6. Susceptibility of strains producing PBP1b variants to cephalexin across pH conditions. Supports Figure 6E. Presents median minimum inhibitory concentrations of cephalexin to MG1655 and PBP1b derivatives across pH conditions (n = 3). Values are represented as μg/mL.

Supplementary file 7. Representative script used to analyze bacterial growth rate datasets. Supports Figure 1 and Figure 1—figure supplement 1. This sample script uses source data from Figure 1—source data 2.

Transparent reporting form

## References

[bib1] Amaral L, Lee Y, Schwarz U, Lorian V (1986). Penicillin-binding site on the *Escherichia coli* cell envelope. Journal of Bacteriology.

[bib2] Auer GK, Lee TK, Rajendram M, Cesar S, Miguel A, Huang KC, Weibel DB (2016). Mechanical genomics identifies diverse modulators of bacterial cell stiffness. Cell Systems.

[bib3] Baba T, Ara T, Hasegawa M, Takai Y, Okumura Y, Baba M, Datsenko KA, Tomita M, Wanner BL, Mori H (2006). Construction of *Escherichia coli* K-12 in-frame, single-gene knockout mutants: the keio collection. Molecular Systems Biology.

[bib4] Banzhaf M, van den Berg van Saparoea B, Terrak M, Fraipont C, Egan A, Philippe J, Zapun A, Breukink E, Nguyen-Distèche M, den Blaauwen T, Vollmer W (2012). Cooperativity of peptidoglycan synthases active in bacterial cell elongation. Molecular Microbiology.

[bib5] Berger-Bächi B (1999). Genetic basis of methicillin resistance in *Staphylococcus aureus*. Cellular and Molecular Life Sciences.

[bib6] Bertsche U, Breukink E, Kast T, Vollmer W (2005). In vitro murein peptidoglycan synthesis by dimers of the bifunctional transglycosylase-transpeptidase PBP1B from *Escherichia coli*. Journal of Biological Chemistry.

[bib7] Bertsche U, Kast T, Wolf B, Fraipont C, Aarsman ME, Kannenberg K, von Rechenberg M, Nguyen-Distèche M, den Blaauwen T, Höltje JV, Vollmer W (2006). Interaction between two murein (peptidoglycan) synthases, PBP3 and PBP1B, in *Escherichia coli*. Molecular Microbiology.

[bib8] Biboy J, Bui NK, Vollmer W (2013). In vitro peptidoglycan synthesis assay with lipid II substrate. Methods in Molecular Biology.

[bib9] Bilobrov VM, Chugaj AV, Bessarabov VI (1990). Urine pH variation dynamics in healthy individuals and stone formers. Urologia Internationalis.

[bib10] Born P, Breukink E, Vollmer W (2006). In vitro synthesis of cross-linked murein and its attachment to sacculi by PBP1A from *Escherichia coli*. Journal of Biological Chemistry.

[bib11] Breukink E, van Heusden HE, Vollmerhaus PJ, Swiezewska E, Brunner L, Walker S, Heck AJ, de Kruijff B (2003). Lipid II is an intrinsic component of the pore induced by nisin in bacterial membranes. Journal of Biological Chemistry.

[bib12] Castanheira S, Cestero JJ, Rico-Pérez G, García P, Cava F, Ayala JA, Pucciarelli MG, García-Del Portillo F (2017). A specialized peptidoglycan synthase promotes *Salmonella* Cell Division inside Host Cells. mBio.

[bib13] Castanie-Cornet MP, Penfound TA, Smith D, Elliott JF, Foster JW (1999). Control of acid resistance in *Escherichia coli*. Journal of Bacteriology.

[bib14] Castanié-Cornet MP, Cam K, Bastiat B, Cros A, Bordes P, Gutierrez C (2010). Acid stress response in *Escherichia coli*: mechanism of regulation of gadA transcription by RcsB and GadE. Nucleic Acids Research.

[bib15] Chan LC, Gilbert A, Basuino L, da Costa TM, Hamilton SM, dos Santos KR, Chambers HF, Chatterjee SS (2016). PBP 4 mediates High-Level resistance to New-Generation cephalosporins in *Staphylococcus aureus*. Antimicrobial Agents and Chemotherapy.

[bib16] Chen SL, Hung CS, Xu J, Reigstad CS, Magrini V, Sabo A, Blasiar D, Bieri T, Meyer RR, Ozersky P, Armstrong JR, Fulton RS, Latreille JP, Spieth J, Hooton TM, Mardis ER, Hultgren SJ, Gordon JI (2006). Identification of genes subject to positive selection in Uropathogenic strains of *Escherichia coli*: a comparative genomics approach. PNAS.

[bib17] Cho H, Wivagg CN, Kapoor M, Barry Z, Rohs PDA, Suh H, Marto JA, Garner EC, Bernhardt TG (2016). Bacterial cell wall biogenesis is mediated by SEDS and PBP polymerase families functioning semi-autonomously. Nature Microbiology.

[bib18] Clark DJ, Maaløe O (1967). DNA replication and the division cycle in *Escherichia coli*. Journal of Molecular Biology.

[bib19] Egan AJ, Jean NL, Koumoutsi A, Bougault CM, Biboy J, Sassine J, Solovyova AS, Breukink E, Typas A, Vollmer W, Simorre JP (2014). Outer-membrane lipoprotein LpoB spans the periplasm to stimulate the peptidoglycan synthase PBP1B. PNAS.

[bib20] Egan AJF, Biboy J, van't Veer I, Breukink E, Vollmer W (2015). Activities and regulation of peptidoglycan synthases. Philosophical Transactions of the Royal Society B: Biological Sciences.

[bib21] Egan AJF, Maya-Martinez R, Ayala I, Bougault CM, Banzhaf M, Breukink E, Vollmer W, Simorre JP (2018). Induced conformational changes activate the peptidoglycan synthase PBP1B. Molecular Microbiology.

[bib22] Egan AJ, Vollmer W (2016). Continuous fluorescence assay for peptidoglycan glycosyltransferases. Methods in Molecular Biology.

[bib23] Ersoy SC, Heithoff DM, Barnes L, Tripp GK, House JK, Marth JD, Smith JW, Mahan MJ (2017). Correcting a fundamental flaw in the paradigm for antimicrobial susceptibility testing. EBioMedicine.

[bib24] García del Portillo F, de Pedro MA (1990). Differential effect of mutational impairment of penicillin-binding proteins 1A and 1B on *Escherichia coli* strains harboring thermosensitive mutations in the cell division genes ftsA, ftsQ, ftsZ, and pbpB. Journal of Bacteriology.

[bib25] Goodell EW, Lopez R, Tomasz A (1976). Suppression of lytic effect of beta lactams on *Escherichia coli* and other bacteria. PNAS.

[bib26] Gray AN, Egan AJF, van't Veer IL, Verheul J, Colavin A, Koumoutsi A, Biboy J, Altelaar AFM, Damen MJ, Huang KC, Simorre J-P, Breukink E, den Blaauwen T, Typas A, Gross CA, Vollmer W (2015). Coordination of peptidoglycan synthesis and outer membrane constriction during *Escherichia coli* cell division. eLife.

[bib27] Heidrich C, Templin MF, Ursinus A, Merdanovic M, Berger J, Schwarz H, de Pedro MA, Höltje JV (2001). Involvement of N-acetylmuramyl-L-alanine amidases in cell separation and antibiotic-induced autolysis of *Escherichia coli*. Molecular Microbiology.

[bib28] Heidrich C, Ursinus A, Berger J, Schwarz H, Höltje JV (2002). Effects of multiple deletions of murein hydrolases on viability, septum cleavage, and sensitivity to large toxic molecules in *Escherichia coli*. Journal of Bacteriology.

[bib29] Henderson LJ, Palmer WW (1912). On the intensity of urinary acidity in normal and pathological conditions. The Journal of Biological Chemistry.

[bib30] Hocking J, Priyadarshini R, Takacs CN, Costa T, Dye NA, Shapiro L, Vollmer W, Jacobs-Wagner C (2012). Osmolality-dependent relocation of penicillin-binding protein PBP2 to the division site in caulobacter crescentus. Journal of Bacteriology.

[bib31] Hugonnet J-E, Mengin-Lecreulx D, Monton A, den Blaauwen T, Carbonnelle E, Veckerlé C, Brun Yves, V., van Nieuwenhze M, Bouchier C, Tu K, Rice LB, Arthur M (2016). Factors essential for L,D-transpeptidase-mediated peptidoglycan cross-linking and β-lactam resistance in *Escherichia coli*. eLife.

[bib32] Ingraham JL, Marr AJ (1996). Effect of temperature, pressure, pH, and osmotic stress on growth. Escherichia Coli And Salmonella Typhimurium: Cellular and Molecular Biology.

[bib33] Jean NL, Bougault CM, Lodge A, Derouaux A, Callens G, Egan AJ, Ayala I, Lewis RJ, Vollmer W, Simorre JP (2014). Elongated structure of the outer-membrane activator of peptidoglycan synthesis LpoA: implications for PBP1A stimulation. Structure.

[bib34] Jensen JB, Peters NK, Bhuvaneswari TV (2002). Redundancy in Periplasmic binding protein-dependent transport systems for trehalose, sucrose, and maltose in sinorhizobium meliloti. Journal of Bacteriology.

[bib35] Jordan KN, Oxford L, O'Byrne CP (1999). Survival of low-pH stress by *Escherichia coli* O157:h7: correlation between alterations in the cell envelope and increased acid tolerance. Applied and Environmental Microbiology.

[bib36] Kocaoglu O, Carlson EE (2015). Profiling of β-Lactam selectivity for Penicillin-Binding proteins in *Escherichia coli* strain DC2. Antimicrobial Agents and Chemotherapy.

[bib37] Krulwich TA, Sachs G, Padan E (2011). Molecular aspects of bacterial pH sensing and homeostasis. Nature Reviews Microbiology.

[bib38] Lai GC, Cho H, Bernhardt TG (2017). The mecillinam resistome reveals a role for peptidoglycan endopeptidases in stimulating cell wall synthesis in *Escherichia coli*. PLOS Genetics.

[bib39] Leclercq S, Derouaux A, Olatunji S, Fraipont C, Egan AJ, Vollmer W, Breukink E, Terrak M (2017). Interplay between Penicillin-binding proteins and SEDS proteins promotes bacterial cell wall synthesis. Scientific Reports.

[bib40] Lommatzsch J, Templin MF, Kraft AR, Vollmer W, Höltje JV (1997). Outer membrane localization of murein hydrolases: mlta, a third lipoprotein lytic transglycosylase in *Escherichia coli*. Journal of Bacteriology.

[bib41] Lu P, Ma D, Chen Y, Guo Y, Chen GQ, Deng H, Shi Y (2013). L-glutamine provides acid resistance for *Escherichia coli* through enzymatic release of ammonia. Cell Research.

[bib42] Lupoli TJ, Lebar MD, Markovski M, Bernhardt T, Kahne D, Walker S (2014). Lipoprotein activators stimulate *Escherichia coli* penicillin-binding proteins by different mechanisms. Journal of the American Chemical Society.

[bib43] Magnet S, Bellais S, Dubost L, Fourgeaud M, Mainardi JL, Petit-Frère S, Marie A, Mengin-Lecreulx D, Arthur M, Gutmann L (2007). Identification of the L,D-transpeptidases responsible for attachment of the braun lipoprotein to *Escherichia coli* peptidoglycan. Journal of Bacteriology.

[bib44] McDonald C, Inohara N, Nuñez G (2005). Peptidoglycan signaling in innate immunity and inflammatory disease. Journal of Biological Chemistry.

[bib45] Meeske AJ, Riley EP, Robins WP, Uehara T, Mekalanos JJ, Kahne D, Walker S, Kruse AC, Bernhardt TG, Rudner DZ (2016). SEDS proteins are a widespread family of bacterial cell wall polymerases. Nature.

[bib46] Meisel U, Höltje JV, Vollmer W (2003). Overproduction of inactive variants of the murein synthase PBP1B causes lysis in *Escherichia coli*. Journal of Bacteriology.

[bib47] Modell JW, Kambara TK, Perchuk BS, Laub MT (2014). A DNA damage-induced, SOS-independent checkpoint regulates cell division in caulobacter crescentus. PLOS Biology.

[bib48] Montón Silva A, Otten C, Biboy J, Breukink E, VanNieuwenhze M, Vollmer W, den Blaauwen T (2018). The fluorescent D-Amino acid NADA as a tool to study the conditional activity of transpeptidases in *Escherichia coli*. Frontiers in Microbiology.

[bib49] Morè N, Martorana AM, Biboy J, Otten C, Winkle M, Serrano CKG, Montón Silva A, Atkinson L, Yau H, Breukink E, den Blaauwen T, Vollmer W, Polissi A (2019). Peptidoglycan remodeling enables *Escherichia coli* to survive severe outer membrane assembly defect. mBio.

[bib50] Müller P, Ewers C, Bertsche U, Anstett M, Kallis T, Breukink E, Fraipont C, Terrak M, Nguyen-Distèche M, Vollmer W (2007). The essential cell division protein FtsN interacts with the murein (peptidoglycan) synthase PBP1B in *Escherichia coli*. Journal of Biological Chemistry.

[bib51] Nelson DE, Young KD (2000). Penicillin binding protein 5 affects cell diameter, contour, and morphology of *Escherichia coli*. Journal of Bacteriology.

[bib52] Nichols RJ, Sen S, Choo YJ, Beltrao P, Zietek M, Chaba R, Lee S, Kazmierczak KM, Lee KJ, Wong A, Shales M, Lovett S, Winkler ME, Krogan NJ, Typas A, Gross CA (2011). Phenotypic landscape of a bacterial cell. Cell.

[bib53] Paradis-Bleau C, Markovski M, Uehara T, Lupoli TJ, Walker S, Kahne DE, Bernhardt TG (2010). Lipoprotein cofactors located in the outer membrane activate bacterial cell wall polymerases. Cell.

[bib54] Paradis-Bleau C, Kritikos G, Orlova K, Typas A, Bernhardt TG (2014). A genome-wide screen for bacterial envelope biogenesis mutants identifies a novel factor involved in cell wall precursor metabolism. PLOS Genetics.

[bib55] Pazos M, Peters K, Vollmer W (2017). Robust peptidoglycan growth by dynamic and variable multi-protein complexes. Current Opinion in Microbiology.

[bib56] Peters JM, Colavin A, Shi H, Czarny TL, Larson MH, Wong S, Hawkins JS, Lu CHS, Koo BM, Marta E, Shiver AL, Whitehead EH, Weissman JS, Brown ED, Qi LS, Huang KC, Gross CA (2016a). A comprehensive, CRISPR-based functional analysis of essential genes in bacteria. Cell.

[bib57] Peters K, Kannan S, Rao VA, Biboy J, Vollmer D, Erickson SW, Lewis RJ, Young KD, Vollmer W (2016b). The redundancy of peptidoglycan carboxypeptidases ensures robust cell shape maintenance in *Escherichia coli*. mBio.

[bib58] Peters K, Pazos M, Edoo Z, Hugonnet JE, Martorana AM, Polissi A, VanNieuwenhze MS, Arthur M, Vollmer W (2018). Copper inhibits peptidoglycan LD-transpeptidases suppressing β-lactam resistance due to bypass of penicillin-binding proteins. PNAS.

[bib59] Pisabarro AG, de Pedro MA, Vázquez D (1985). Structural modifications in the peptidoglycan of *Escherichia coli* associated with changes in the state of growth of the culture. Journal of Bacteriology.

[bib60] Ranjit DK, Jorgenson MA, Young KD (2017). PBP1B Glycosyltransferase and Transpeptidase Activities Play Different Essential Roles during the *De Novo* Regeneration of Rod Morphology in *Escherichia coli*. Journal of Bacteriology.

[bib61] Rizzitello AE, Harper JR, Silhavy TJ (2001). Genetic evidence for parallel pathways of chaperone activity in the periplasm of *Escherichia coli*. Journal of Bacteriology.

[bib62] Rosenbusch JP (1990). Structural and functional properties of porin channels in *E. coli* outer membranes. Experientia.

[bib63] Sarkar SK, Chowdhury C, Ghosh AS (2010). Deletion of penicillin-binding protein 5 (PBP5) sensitises *Escherichia coli* cells to beta-lactam agents. International Journal of Antimicrobial Agents.

[bib64] Sauvage E, Kerff F, Terrak M, Ayala JA, Charlier P (2008). The penicillin-binding proteins: structure and role in peptidoglycan biosynthesis. FEMS Microbiology Reviews.

[bib65] Schiffer G, Höltje JV (1999). Cloning and characterization of PBP 1C, a third member of the multimodular class A penicillin-binding proteins of *Escherichia coli*. Journal of Biological Chemistry.

[bib66] Schindelin J, Arganda-Carreras I, Frise E, Kaynig V, Longair M, Pietzsch T, Preibisch S, Rueden C, Saalfeld S, Schmid B, Tinevez JY, White DJ, Hartenstein V, Eliceiri K, Tomancak P, Cardona A (2012). Fiji: an open-source platform for biological-image analysis. Nature Methods.

[bib67] Schmidt LS, Botta G, Park JT (1981). Effects of furazlocillin, a beta-lactam antibiotic which binds selectively to penicillin-binding protein 3, on *Escherichia coli* mutants deficient in other penicillin-binding proteins. Journal of Bacteriology.

[bib68] Schmidt A, Kochanowski K, Vedelaar S, Ahrné E, Volkmer B, Callipo L, Knoops K, Bauer M, Aebersold R, Heinemann M (2016). The quantitative and condition-dependent *Escherichia coli* proteome. Nature Biotechnology.

[bib69] Singh SK, SaiSree L, Amrutha RN, Reddy M (2012). Three redundant murein endopeptidases catalyse an essential cleavage step in peptidoglycan synthesis of *Escherichia coli* K12. Molecular Microbiology.

[bib70] Slonczewski JL, Rosen BP, Alger JR, Macnab RM (1981). pH homeostasis in *Escherichia coli*: measurement by 31P nuclear magnetic resonance of methylphosphonate and phosphate. PNAS.

[bib71] Smith HE, Blair JM (2014). Redundancy in the periplasmic adaptor proteins AcrA and AcrE provides resilience and an ability to export substrates of multidrug efflux. Journal of Antimicrobial Chemotherapy.

[bib72] Stylianidou S, Brennan C, Nissen SB, Kuwada NJ, Wiggins PA (2016). *SuperSegger*: robust image segmentation, analysis and lineage tracking of bacterial cells. Molecular Microbiology.

[bib73] Suzuki H, Nishimura Y, Hirota Y (1978). On the process of cellular division in *Escherichia coli*: a series of mutants of *E. coli* altered in the penicillin-binding proteins. PNAS.

[bib74] Taguchi A, Welsh MA, Marmont LS, Lee W, Sjodt M, Kruse AC, Kahne D, Bernhardt TG, Walker S (2019). FtsW is a peptidoglycan polymerase that is functional only in complex with its cognate penicillin-binding protein. Nature Microbiology.

[bib75] Terrak M, Ghosh TK, van Heijenoort J, Van Beeumen J, Lampilas M, Aszodi J, Ayala JA, Ghuysen JM, Nguyen-Distèche M (1999). The catalytic, glycosyl transferase and acyl transferase modules of the cell wall peptidoglycan-polymerizing penicillin-binding protein 1b of *Escherichia coli*. Molecular Microbiology.

[bib76] Thulin E, Thulin M, Andersson DI (2017). Reversion of High-level mecillinam resistance to susceptibility in *Escherichia coli* during growth in urine. EBioMedicine.

[bib77] Typas A, Banzhaf M, van den Berg van Saparoea B, Verheul J, Biboy J, Nichols RJ, Zietek M, Beilharz K, Kannenberg K, von Rechenberg M, Breukink E, den Blaauwen T, Gross CA, Vollmer W (2010). Regulation of peptidoglycan synthesis by outer-membrane proteins. Cell.

[bib78] Typas A, Banzhaf M, Gross CA, Vollmer W (2012). From the regulation of peptidoglycan synthesis to bacterial growth and morphology. Nature Reviews Microbiology.

[bib79] van Straaten KE, Dijkstra BW, Vollmer W, Thunnissen AM (2005). Crystal structure of MltA from *Escherichia coli* reveals a unique lytic transglycosylase fold. Journal of Molecular Biology.

[bib80] Vollmer W, Blanot D, de Pedro MA (2008). Peptidoglycan structure and architecture. FEMS Microbiology Reviews.

[bib81] Watson BW, Meldrum SJ, Riddle HC, Brown RL, Sladen GE (1972). pH profile of gut as measured by radiotelemetry capsule. Bmj.

[bib82] Wilks JC, Kitko RD, Cleeton SH, Lee GE, Ugwu CS, Jones BD, BonDurant SS, Slonczewski JL (2009). Acid and base stress and transcriptomic responses in *Bacillus subtilis*. Applied and Environmental Microbiology.

[bib83] Wilks JC, Slonczewski JL (2007). pH of the cytoplasm and periplasm of *Escherichia coli*: rapid measurement by green fluorescent protein fluorimetry. Journal of Bacteriology.

[bib84] Yousif SY, Broome-Smith JK, Spratt BG (1985). Lysis of *Escherichia coli* by beta-lactam antibiotics: deletion analysis of the role of penicillin-binding proteins 1A and 1B. Journal of General Microbiology.

