# Supplementary material for: Plasticity of Escherichia coli cell wall metabolism promotes fitness and antibiotic resistance across environmental conditions
Source: eLife. 2019 Apr 9;8:e40754. doi: 10.7554/eLife.40754 (PMC6456298; doi:10.7554/eLife.40754)
Supplement: Supplementary file 1. [file elife-40754-supp1.docx]

**Supplementary File 1.** Bacterial strains used in this study.

| **Strain ID** | **Genotype** | **Source**^a^ | **Keio Source ID^b^** |
| --- | --- | --- | --- |
| MG1655 | *rph1* *ilvG* *rfb*-50 | (1) |  |
| UTI89 |  | (2) |  |
| EAM546 | MG1655 *mrcB*::*kan* | P1(JW0145-1) x MG1655 | JW0145-1 |
| EAM696 | MG1655 *mrcB*::*frt* | EAM546/pCP20 |  |
| EAM543 | MG1655 *mrcA*::*kan* | P1(JW3359-1) x MG1655 | JW3359-1 |
| EAM899 | MG1655 *mrcA*::*frt* | EAM543/pCP20 |  |
| EAM694 | MG1655 *pbpC*::*kan* | P1(JW2503-1) x MG1655 | JW2503-1 |
| DZ108 | MG1655 *dacA*::*kan* | P1(JW0627-1) x MG1655 | JW0627-1 |
| DZ110 | MG1655 *dacB*::*kan* | P1(JW3149-1) x MG1655 | JW3149-1 |
| EAM794 | MG1655 *yfeW*::*kan* | P1(JW5395-1) x MG1655 | JW5395-1 |
| DZ130 | MG1655 *dacC*::*kan* | P1(JW0823-1) x MG1655 | JW0823-1 |
| EAM944 | MG1655 *dacD*::*kan* | P1(JW5329-1) x MG1655 | JW5329-1 |
| EAM759 | MG1655 *amiA*::*kan* | P1(JW2428-1) x MG1655 | JW2428-1 |
| EAM946 | MG1655 *amiB*::*kan* | P1(JW4127-1) x MG1655 | JW4127-1 |
| EAM763 | MG1655 *amiC*::*kan* | P1(JW5449-1) x MG1655 | JW5449-1 |
| EAM948 | MG1655 *amiD*::*kan* | P1(JW0581-1) x MG1655 | JW0581-1 |
| EAM790 | MG1655 *mltA*::*kan* | P1(JW2784-1) x MG1655 | JW2784-1 |
| EAM792 | MG1655 *mltB*::*kan* | P1(JW2671-1) x MG1655 | JW2671-1 |
| EAM800 | MG1655 *mltC*::*kan* | P1(JW5481-1) x MG1655 | JW5481-1 |
| EAM802 | MG1655 *mltD*::*kan* | P1(JW5018-1) x MG1655 | JW5018-1 |
| EAM804 | MG1655 *emtA*::*kan* | P1(JW5821-1) x MG1655 | JW5821-1 |
| EAM895 | MG1655 *mltF*::*kan* | P1(JW2542-6) x MG1655 | JW2542-6 |
| EAM798 | MG1655 *yceG*::*kan* | P1(JW1083-1) x MG1655 | JW1083-1 |
| EAM841 | MG1655 *slt*::*kan* | P1(JW4355-1) x MG1655 | JW4355-1 |
| EAM893 | MG1655 *rlpA*::*kan* | P1(JW0628-1) x MG1655 | JW0628-1 |
| EAM814 | MG1655 *ydho*::*kan* | P1(JW5270-1) x MG1655 | JW5270-1 |
| EAM1032 | MG1655 *spr*::*kan* | P1(JW2163-1) x MG1655 | JW2163-1 |
| EAM818 | MG1655 *mepA*::*kan* | P1(JW2325-1) x MG1655 | JW2325-1 |
| EAM820 | MG1655 *pbpG*::*kan* | P1(JW5355-1) x MG1655 | JW5355-1 |
| EAM816 | MG1655 *yebA*::*kan* | P1(JW5304-1) x MG1655 | JW5304-1 |
| EAM663 | MG1655 *ycfS*::*kan* | P1(JW5820-1) x MG1655 | JW5820-2 |
| EAM665 | MG1655 *ybiS*::*kan* | P1(JW0803-1) x MG1655 | JW0803-1 |
| EAM669 | MG1655 *erfK*::*kan* | P1(JW1968-1) x MG1655 | JW1968-1 |
| EAM667 | MG1655 *ynhG*::*kan* | P1(JW1668-1) x MG1655 | JW1668-1 |
| EAM671 | MG1655 *ycbB*::*kan* | P1(JW0908-1) x MG1655 | JW0908-1 |
| EAM897 | MG1655 *yafK*::*kan* | P1(JW0214-1) x MG1655 | JW0214-1 |
| EAM657 | MG1655 *lpoA*::*kan* | P1(JW3116-1) x MG1655 | JW3116-1 |
| EAM659 | MG1655 *lpoB*::*kan* | P1(JW5157-1) x MG1655 | JW5157-1 |
| EAM718 | MG1655 *mrcB::kan attHK022 plac::gfp-mrcB*(𝛾) | (3) |  |
| EAM707 | MG1655 *mrcA::kan attHK022 plac::gfp-mrcA* | (3) |  |
| EAM749 | MG1655 *ampC::kan* | P1(JW4111-2) x MG1655 | JW4111-2 |
| BW25113∆6LDT | BW25113 *ldtA::frt ldtB::frt ldtC::frt, ldtD::frt ldtE::frt ldtF::frt* | (4) |  |
| EAM1152 | MG1655 *ynhG::frt ycbB::frt yafK::kan* | P1(JW0214-1) x EAM1151 | JW0214-1 |
| EAM1137 | MG1655 *mrcA::frt pbpC::kan* | P1(JW2503-1) x EAM899 | JW2503-1 |
| EAM1139 | MG1655 *mrcB::frt pbpC::kan* | P1(JW2503-1) x EAM696 | JW2503-1 |
| EAM831 | MG1655 *tolA::kan* | P1(JW0729-3) x MG1655 | JW0729-3 |

^a^Strains constructed by P1 transduction from kanamycin insertional deletions from the Keio collection are described using the shorthand: P1(donor) x recipient. Strains constructed from the removal of a Kan^R^ cassette using pCP20 are indicated as: Parental strain/pCP20 (5).

^b^Strains sourced from the Coli Genetic Stock Center (6)
