# Supplementary material for: Plasticity of Escherichia coli cell wall metabolism promotes fitness and antibiotic resistance across environmental conditions
Source: eLife. 2019 Apr 9;8:e40754. doi: 10.7554/eLife.40754 (PMC6456298; doi:10.7554/eLife.40754)
Supplement: Supplementary file 2. [file elife-40754-supp2.docx]

**Supplementary File 2.** Plasmids used in this study.

| **Annotation** | **Genotype** | **Source** |
| --- | --- | --- |
| pCP20 | *bla* *cat* *cI*857 *repA*(ts) P_R_::*flp* | (5) |
| pUM1Bα | *Plac::mrcBα* | (7) |
| pUM1B*γ* | *Plac::mrcBγ* | (7) |
| pUM1Bα* | *Plac*::*mrcBα(*S510A) | (7) |
| pUM1BTG*α | *Plac*::*mrcBα*(E233Q) | (7) |
| pUM1BTG*α* | *Plac*::*mrcBα(*S510A/E233Q) | (7) |
| pBH234 | *Plac::gfp-mut3* | (8) |

**References**

1. Guyer MS, Reed RR, Steitz JA, Low KB. Identification of a sex-factor-affinity site in E. coli as gamma delta. Cold Spring Harb Symp Quant Biol. 1981;45 Pt 1:135–40.

2. Mulvey MA, Schilling JD, Hultgren SJ. Establishment of a Persistent Escherichia coli Reservoir during the Acute Phase of a Bladder Infection. Infection and Immunity. American Society for Microbiology Journals; 2001 Jul 1;69(7):4572–9.

3. Paradis-Bleau C, Markovski M, Uehara T, Lupoli TJ, Walker S, Kahne DE, et al. Lipoprotein cofactors located in the outer membrane activate bacterial cell wall polymerases. Cell. 2010 Dec 23;143(7):1110–20.

4. Kuru E, Lambert C, Rittichier J, Till R, Ducret A, Derouaux A, et al. Fluorescent D -amino-acids reveal bi-cellular cell wall modifications important for Bdellovibrio bacteriovorus predation. Nat Microbiol. Nature Publishing Group; 2017 Dec 1;2(12):1648–57.

5. Datsenko KA, Wanner BL. One-step inactivation of chromosomal genes in Escherichia coli K-12 using PCR products. Proceedings of the National Academy of Sciences. National Academy of Sciences; 2000 Jun 6;97(12):6640–5.

6. Baba T, Ara T, Hasegawa M, Takai Y, Okumura Y, Baba M, et al. Construction of Escherichia coli K-12 in-frame, single-gene knockout mutants: the Keio collection. Molecular Systems Biology. EMBO Press; 2006 Feb 21;2(1):473.

7. Meisel U, Höltje J-V, Vollmer W. Overproduction of inactive variants of the murein synthase PBP1B causes lysis in Escherichia coli. J Bacteriol. American Society for Microbiology (ASM); 2003 Sep;185(18):5342–8.

8. Bardill JP, Miller JL, Vogel JP. IcmS‐dependent translocation of SdeA into macrophages by the Legionella pneumophila type IV secretion system. Mol Microbiol. John Wiley & Sons, Ltd (10.1111); 2005 Apr 1;56(1):90–103.
