# Supplementary material for: Plasticity of Escherichia coli cell wall metabolism promotes fitness and antibiotic resistance across environmental conditions
Source: eLife. 2019 Apr 9;8:e40754. doi: 10.7554/eLife.40754 (PMC6456298; doi:10.7554/eLife.40754)
Supplement: Supplementary file 3. — Supports Figure 1. Presents mean mass doubling time ± standard deviation of each cell wall mutant at pH 4.8, 6.9, and 8.2 during preliminary screen (n = 3). [file elife-40754-supp3.docx]

**Supplementary File 3.** Summary of growth rate screen.

|  | | **Mass doublings per hour^a^** | | | |
| --- | --- | --- | --- | --- | --- |
|  |  | **pH** | | | |
| **Mutant Genotype** | **Strain ID** | **4.8** | **6.9** | **8.2** | |
| WT | MG1655 | 1.54 +/- 0.02 | 2.49 +/- 0.06 | 2.10 +/- 0.05 | |
| ∆*mrcA* | EAM543 | 1.43 +/- 0.08 | 2.30 +/- 0.03 | 1.90 +/- 0.03 | |
| ∆*mrcB* | EAM546 | 0 | 2.45 +/- 0.02 | 2.12 +/- 0.05 | |
| ∆*pbpC* | EAM694 | 1.48 +/- 0.01 | 2.47 +/- 0.01 | 2.09 +/- 0.03 | |
| ∆*dacB* | DZ110 | 1.45 +/- 0.05 | 2.43 +/- 0.08 | 2.05 +/- 0.01 | |
| ∆*yfeW* | EAM794 | 1.45 +/- 0.02 | 2.53 +/- 0.09 | 2.09 +/- 0.05 | |
| ∆*dacA* | DZ108 | 1.45 +/- 0.06 | 2.32 +/- 0.13 | 2.07 +/- 0.03 | |
| ∆*dacC* | DZ130 | 1.52 +/- 0.03 | 2.44 +/- 0.03 | 2.11 +/- 0.08 | |
| ∆*dacD* | EAM773 | 1.58 +/- 0.02 | 2.63 +/- 0.04 | 2.31 +/- 0.07 | |
| ∆*amiA* | EAM759 | 1.42 +/- 0.05 | 2.54 +/- 0.08 | 2.02 +/- 0.05 | |
| ∆*amiB* | EAM761 | 1.61 +/- 0.07 | 2.62 +/- 0.06 | 2.08 +/- 0.05 | |
| ∆*amiC* | EAM763 | 1.51 +/- 0.01 | 2.48 +/- 0.07 | 2.02 +/- 0.06 | |
| ∆*amiD* | EAM765 | 1.62 +/- 0.05 | 2.63 +/- 0.04 | 2.29 +/- 0.06 | |
| ∆*slt* | EAM841 | 1.52 +/- 0.04 | 2.52 +/- 0.10 | 2.00 +/- 0.01 | |
| ∆*mltA* | EAM790 | 1.35 +/- 0.03 | 2.42 +/- 0.11 | 2.01 +/- 0.18 | |
| ∆*mltB* | EAM792 | 1.44 +/- 0.01 | 2.48 +/- 0.12 | 2.01 +/- 0.04 | |
| ∆*mltC* | EAM800 | 1.35 +/- 0.20 | 2.47 +/- 0.07 | 2.06 +/- 0.07 | |
| ∆*mltD* | EAM802 | 1.48 +/- 0.01 | 2.53 +/- 0.06 | 2.15 +/- 0.01 | |
| ∆*emtA* | EAM804 | 1.47 +/- 0.03 | 2.51 +/- 0.10 | 2.04 +/- 0.02 | |
| ∆*mltF* | EAM895 | 1.60 +/- 0.04 | 2.54 +/- 0.08 | 2.11 +/- 0.05 | |
| ∆*yceG* | EAM798 | 1.56 +/- 0.03 | 2.21 +/- 0.03 | 1.64 +/- 0.06 | |
| ∆*rlpA* | EAM893 | 1.60 +/- 0.10 | 2.56 +/- 0.02 | 2.13 +/- 0.06 | |
| ∆*ydhO* | EAM814 | 1.64 +/- 0.00 | 2.42 +/- 0.04 | 2.07 +/- 0.06 | |
| ∆*spr* | EAM840 | 1.40 +/- 0.01 | 2.56 +/- 0.04 | 2.17 +/- 0.1 | |
| ∆*mepA* | EAM818 | 1.57 +/- 0.01 | 2.57 +/- 0.04 | 2.16 +/- 0.04 | |
| ∆*pbpG* | EAM820 | 1.56 +/- 0.01 | 2.53 +/- 0.04 | 2.14 +/- 0.01 | |
| ∆*yebA* | EAM816 | 1.62 +/- 0.03 | 2.38 +/- 0.05 | 2.04 +/- 0.04 | |
| ∆*ycfS* | EAM663 | 1.50 +/- 0.02 | 2.44 +/- 0.03 | 2.19 +/- 0.04 | |
| ∆*ybiS* | EAM665 | 1.52 +/- 0.06 | 2.44 +/- 0.03 | 2.17 +/- 0.01 | |
| ∆*erfK* | EAM669 | 1.45 +/0 0.04 | 2.44 +/- 0.07 | 2.05 +/- 0.01 | |
| ∆*ycbB* | EAM671 | 1.43 +/- 0.05 | 2.47 +/- 0.05 | 2.15 +/- 0.01 | |
| ∆*ynhG* | EAM667 | 1.49 +/- 0.02 | 2.34 +/- 0.12 | 2.11 +/- 0.03 | |
| ∆*yafK* | EAM897 | 1.53 +/- 0.05 | 2.66 +/- 0.01 | 2.08 +/- 0.05 | |
| ^a^Growth rate values are represented as average mass doublings per hour +/- standard deviation of three biological replicates per mutant per pH condition. | | | | |  |
