# Supplementary material for: Plasticity of Escherichia coli cell wall metabolism promotes fitness and antibiotic resistance across environmental conditions
Source: eLife. 2019 Apr 9;8:e40754. doi: 10.7554/eLife.40754 (PMC6456298; doi:10.7554/eLife.40754)
Supplement: Supplementary file 4. — Supports Figure 6A. Presents median minimum inhibitory concentrations of indicated β-lactam antibiotics to MG1655 across pH conditions of at least three biological replicates. Values are represented as μg/mL. [file elife-40754-supp4.docx]

**Supplementary File 4.** β-lactam sensitivity of MG1655 across pH conditions.

| **Antibiotic** | **MIC (μg/mL) at indicated pH** | | | | | | | |
| --- | --- | --- | --- | --- | --- | --- | --- | --- |
|  | **4.5** | **5.0** | **5.5** | **6.0** | **6.5** | **7.0** | **7.5** | **8.0** |
| AMP | 8.0 | 8.0 | 8.0 | 8.0 | 8.0 | 8.0 | 8.0 | 16.0 |
| AMX | 500 | 500 | 250 | 250 | 250 | 250 | 250 | 250 |
| CFS | 62.5 | 62.5 | 31.25 | 62.5 | 62.5 | 62.5 | 62.5 | 62.5 |
| MEC | 12.5 | 1.6 | 0.8 | 0.8 | 0.4 | 0.4 | 3.1 | 1.6 |
| DOR | 1.0 | 0.125 | 0.125 | 0.0625 | 0.0625 | 0.0625 | 0.0625 | 0.03125 |
| MEM | 0.5 | 0.25 | 0.125 | 0.125 | 0.0625 | 0.0625 | 0.0625 | 0.0625 |
| CEX | 100.0 | 50.0 | 50.0 | 25.0 | 12.5 | 12.5 | 12.5 | 12.5 |
| AZT | 0.59 | 0.29 | 0.15 | 0.07 | 0.07 | 0.07 | 0.07 | 0.07 |
| PIP | 3.125 | 6.25 | 6.25 | 3.125 | 1.5625 | 1.5625 | 1.5625 | 1.5625 |
| CH | 1.625 | 3.125 | 6.25 | 6.25 | 6.25 | 6.25 | 6.25 | 6.25 |
