# Supplementary material for: Plasticity of Escherichia coli cell wall metabolism promotes fitness and antibiotic resistance across environmental conditions
Source: eLife. 2019 Apr 9;8:e40754. doi: 10.7554/eLife.40754 (PMC6456298; doi:10.7554/eLife.40754)
Supplement: Supplementary file 5. — Supports Figure 6D. Presents median minimum inhibitory concentrations of cephalexin (CEX) and mecillinam (MEC) to UTI89 across pH conditions in LB and in urine (n = 3). Values are represented as μg/mL. [file elife-40754-supp5.docx]

**Supplementary File 5.** β-lactam sensitivity of UTI89 across pH conditions.

| **Growth media** | **CEX MIC (μg/mL)** | | **MEC (μg/mL)** | |
| --- | --- | --- | --- | --- |
|  | **pH 7.0** | **pH 5.0** | **pH 7.0** | **pH 5.0** |
| LB | 6.25 | 100 | 0.390625 | 1.5625 |
| Urine | 12.5 | 200 | 1.5625 | 12.5 |
