# Supplementary material for: Plasticity of Escherichia coli cell wall metabolism promotes fitness and antibiotic resistance across environmental conditions
Source: eLife. 2019 Apr 9;8:e40754. doi: 10.7554/eLife.40754 (PMC6456298; doi:10.7554/eLife.40754)
Supplement: Supplementary file 6. — Supports Figure 6E. Presents median minimum inhibitory concentrations of cephalexin to MG1655 and PBP1b derivatives across pH conditions (n = 3). Values are represented as μg/mL. [file elife-40754-supp6.docx]

**Supplementary File 6.** Susceptibility of strains producing PBP1b variants to cephalexin across pH conditions.

| **Strain** | **CEX MIC (μg/mL)** | |
| --- | --- | --- |
|  | **pH 7.0** | **pH 5.5** |
| WT (MG1655) | 6.25 | 25.0 |
| ∆*mrcB* (EAM696) | 3.125 | 0.78125 |
| ∆*mrcB* + PBP1bα | 6.25 | 25.0 |
| ∆*mrcB* + PBP1bα(TP*) | 3.125 | 0.78125 |
| ∆*mrcB* + PBP1bα(GT*) | 3.125 | 1.6 |
| ∆*mrcB* + PBP1bα(TP*GT*) | 3.125 | 1.6 |
