# Supplementary material for: Plasticity of Escherichia coli cell wall metabolism promotes fitness and antibiotic resistance across environmental conditions
Source: eLife. 2019 Apr 9;8:e40754. doi: 10.7554/eLife.40754 (PMC6456298; doi:10.7554/eLife.40754)
Supplement: Supplementary file 7. — Supports Figure 1 and Figure 1—figure supplement 1. This sample script uses source data from Figure 1—source data 2. [file elife-40754-supp7.docx]

**Supplementary File 7. Representative script used to analyze bacterial growth rate datasets.** Supports Figure 1 and Figure 1- Figure Supplement 1. This sample script uses source data from Figure 1 – Source Data 2.

### Representative script used intended to calculate the growth rate of bacterial cultures based on OD600 readings and compare fits.

### This script uses the data for the pH 6.9 growth curves represented in Figure 1 - Figure Supplement 1 (middle panel).

### Set working directory

getwd()

setwd("/Users/eamueller/Desktop/Levin_Lab/Manuscripts/Growth_pH/eLife/Revisions/Growth Rate Fits")

getwd()

### Read in data of .csv file

data <- read.csv("Example_pH6.9.csv")

### Display first part of table

head(data)

### Look at subset of data; plot to visualize

data_WT <- data[,2]

data_WT

time <- data[,1]

time

plot(time, data_WT, xlab = "time(min)", ylab = "OD600" )

### Find min values for each column (column = 2, row = 1)

min <- apply(data,2,min)

min

### Turn min into vector and subtract from each column; plot all curves on single plot

vector <- c(min)

vector

datanorm <- sweep(data, 2, vector)

datanorm

head(datanorm)

jpeg('rplot_pH6.9_full.jpg')

matplot(datanorm[,1],datanorm[,-1], type ='l', lty=1, xlab = 'time (min)', ylab = 'OD600', main = 'pH 6.9', cex.lab=1.5, cex.main=3,lwd = 4)

par(mar=c(7,7,2,2))

legend("topleft", legend = c("WT","pbpC","mrcB","mrcA"), col=1:4, pch= 20)

dev.off()

### Crop columns to only encode values between 0.005-0.1. This is referred to a subsetting the data. Make sure to select the columns that you want to crop.

data_WTnorm <- datanorm[,2]

data_pbpCnorm <- datanorm[,3]

data_mrcBnorm <- datanorm[,4]

data_mrcAnorm <- datanorm[,5]

df <- data.frame(time, data_WTnorm)

WT_subset <- subset(df, data_WTnorm > 0.005 & data_WTnorm <= 0.1, select = c(time, data_WTnorm))

WT_subset

plot(WT_subset$time, WT_subset$data_WTnorm,xlab = "time", ylab = "OD600")

df <- data.frame(time, data_pbpCnorm)

pbpC_subset <- subset(df, data_pbpCnorm > 0.005 & data_pbpCnorm <= 0.1, select = c(time, data_pbpCnorm))

pbpC_subset

plot(pbpC_subset$time, pbpC_subset$data_pbpCnorm,xlab = "time", ylab = "OD600")

df <- data.frame(time, data_mrcBnorm)

mrcB_subset <- subset(df, data_mrcBnorm > 0.005 & data_mrcBnorm <= 0.1, select = c(time, data_mrcBnorm))

mrcB_subset

plot(mrcB_subset$time, mrcB_subset$data_mrcBnorm,xlab = "time", ylab = "OD600")

df <- data.frame(time, data_mrcAnorm)

mrcA_subset <- subset(df, data_mrcAnorm > 0.005 & data_mrcAnorm <= 0.1, select = c(time, data_mrcAnorm))

mrcA_subset

plot(mrcA_subset$time, mrcA_subset$data_mrcAnorm,xlab = "time", ylab = "OD600")

### Fit exponential curve to line via least mean squares fitting and check to see how it fits the data.

fit_WT <- lm(log(WT_subset$data_WTnorm) ~ log(WT_subset$time))

summary(fit_WT)

fit_WT$coefficients

fit_pbpC <- lm(log(pbpC_subset$data_pbpCnorm) ~ log(pbpC_subset$time))

summary(fit_pbpC)

fit_pbpC$coefficients

fit_mrcB <- lm(log(mrcB_subset$data_mrcBnorm) ~ log(mrcB_subset$time))

summary(fit_mrcB)

fit_mrcB$coefficients

fit_mrcA <- lm(log(mrcA_subset$data_mrcAnorm) ~ log(mrcA_subset$time))

summary(fit_mrcA)

fit_mrcA$coefficients

### plot the fitted line on top of the data

timevalues_WT <- seq(390,480,10) ##This creates x values for line

timevalues_pbpC <- seq(370,460,10)

timevalues_mrcB <- seq(400,480,10)

timevalues_mrcA <- seq(390,480,10)

fit_lm2_WT <- exp(predict(fit_WT,list(Time=timevalues_WT)))

fit_lm2_pbpC <- exp(predict(fit_pbpC,list(Time=timevalues_pbpC)))

fit_lm2_mrcB <- exp(predict(fit_mrcB,list(Time=timevalues_mrcB)))

fit_lm2_mrcA <- exp(predict(fit_mrcA,list(Time=timevalues_mrcA)))

jpeg('rplot_pH6.9_fits.jpg')

plot(WT_subset$time, WT_subset$data_WTnorm,xlab = "time (min)", ylab = "OD600", main = "pH 6.9 Cropped", col=1, cex.lab=1.5, cex.main=3)

par(new=T)

lines(timevalues_WT,fit_lm2_WT, lwd=3, col ="black")

points(pbpC_subset$time, pbpC_subset$data_pbpCnorm, main = "pbpC", xlab = "time (min)", ylab = "OD600", col = 2)

par(new=T)

lines(timevalues_pbpC,fit_lm2_pbpC, lwd=3, col ="red")

points(mrcB_subset$time, mrcB_subset$data_mrcBnorm, main = "mrcB", xlab = "time (min)", ylab = "OD600", col = 3)

par(new=T)

lines(timevalues_mrcB,fit_lm2_mrcB, lwd=3, col ="green")

points(mrcA_subset$time, mrcA_subset$data_mrcAnorm, main = "mrcA", xlab = "time (min)", ylab = "OD600", col=4)

par(new=T)

lines(timevalues_mrcA,fit_lm2_mrcA, lwd=3, col ="blue")

legend("topleft", legend = c("WT","pbpC","mrcA"), col= c("black", "red", "blue"), pch= 20)

dev.off()

### Calculate growth rate from equation Afinal = Aintiale^(kt) rearrange to (log(Afinal/Ainitial)/t = k

model_df <- data.frame(timevalues,fit_lm2)

Afinal <- tail(fit_lm2,1)

Ainitial <- head(fit_lm2,1)

tfinal <- tail(timevalues,1)

tinitial <- head(timevalues,1)

k <- (log(Afinal/Ainitial))/(tfinal-tinitial)

k

### Convert k to doubling time from equation dt = log(2)/k

dt = log(2)/k

dt

### Convert doubling time to doubling per hour dph = 60/dt

dph = 60/dt

dph
